# Multiscale artificial spider web for comprehensive pressure sensing and human-machine interaction

**DOI:** 10.1038/s41467-026-74200-y

**Published:** 2026-07-04

**Authors:** Jing Dai, Kwan-Nyeong Kim, Guangzhong Xie, Dong-Su Choi, Seung-Woo Lee, Eojin Yoon, Chan-Yul Park, Han Dai, Somin Kim, Hyun-Haeng Lee, Tae-Woo Lee

**Affiliations:** 1 Department of Materials Science and Engineering, Seoul National University, Seoul, Republic of Korea; 2 School of Optoelectronic Science and Engineering, University of Electronic Science and Technology of China, Chengdu, China; 3 Institute of Art and Art Design, Nanyang Normal University, Nanyang, China; 4 Institute of Engineering Research, Soft Foundry, Research Institute of Advanced Materials, Interdisciplinary Program in Bioengineering, Seoul National University, Seoul, Republic of Korea

**Keywords:** Sensors and biosensors, Polymers, Polymers

## Abstract

Flexible pressure sensors are key components of Internet of Things systems for monitoring environmental and physiological signals, yet simultaneously achieving a high sensitivity, a fast response time, and a high mechanical durability remains challenging owing to the lack of sophisticated structural designs that balance sensing performance and robustness. Here, we demonstrate a multiscale artificial spider web (MASW) fabricated via copper-mesh-assisted electrospinning of biodegradable polylactic acid, forming a nanofiber network that efficiently transmits stress while enhancing mechanical stability. The resulting pressure sensor simultaneously shows a high sensitivity of 39.85 [kPa]^−1^, a fast response of 42 ms and high durability over 6000 loading cycles, enabling reliable and versatile neural-network-assisted real-time monitoring of multiple physiological signals, including pulse, breathing, and vocalization. Furthermore, leveraging precise joint movement signal acquisition, a human-machine interaction system was developed with potential applications in fine motor rehabilitation for Parkinson’s disease, suggesting strong promise for sustainable healthcare and IoT systems.

## Abstract (web summary)

Developing flexible sensors is essential for intelligent healthcare monitoring and robotics, but simultaneously optimizing sensitivity, response time, and mechanical properties remains challenging. Here, the authors report a spider web mimicking polymer nanomesh for complex pressure monitoring tasks.

## Introduction

Recent flexible electronic devices have witnessed rapid development in areas such as wearable healthcare, intelligent robotics, and the next-generation Internet of Things ^1–3^. Among them, flexible pressure sensors, which effectively conform to diverse surfaces, play a pivotal role by converting external mechanical stimuli into readable signals and enabling versatile applications in medical health monitoring, human-computer interaction, and wearable electronics^4,5^.

However, flexible pressure sensors still face a major challenge in simultaneously achieving high sensitivity, fast response, and robust mechanical integrity. This challenge mainly originates from the absence of sophisticated structural designs within pressure-sensing composites, which is essential for efficient stress transmission and structural reliability^6–8^. Although various nanostructural engineering strategies have been introduced to enhance sensitivity and response speed, the interfacial architecture between the sensing components and the flexible matrix in the active layer remains difficult to optimize. Simple planar or randomly distributed structures often result in weak interfacial adhesion, inefficient stress transmission, and mechanical degradation under repeated deformation^9^. Consequently, a persistent trade-off exists between sensing performance and mechanical stability, hindering accurate monitoring of complex physiological signals such as pulse, joint, and laryngeal movements^10,11^. Therefore, developing flexible pressure sensors that can balance high sensitivity, rapid response, and durable mechanical integrity remains an urgent yet unresolved challenge.

In this work, inspired by the architecture of natural spider webs, we propose and construct a flexible pressure sensor that integrates a multiscale conductive three-dimensional (3D) network. The multiscale artificial spider web (MASW) is fabricated via a copper-mesh-assisted electrospinning process of polylactic acid (PLA), where the resulting electrospun fiber network markedly enhances stress transmission while the embedded mesh skeleton reinforces mechanical stability. Furthermore, coating with conductive nanoparticles and nanowires establishes a core-shell-network dual microstructure on the PLA matrix, enabling 3D conductive contact in the sensing layer to significantly enhance sensitivity and response speed. The sensing mechanism is further analyzed based on the principle of contact mechanics, percolation behavior, and tunneling conduction, providing a clear physical understanding of the pressure-dependent electrical response. As a result, the MASW exhibits exceptional overall performance among pressure-resistive sensors, simultaneously achieving ultrahigh sensitivity (39.85 [kPa]^−1^) within 0-100 kPa (The sensitivity far exceeding that of the commercial sensors (~0.05 [kPa]^−1^) for the same pressure range), rapid response time (42 ms), high cyclic stability over 6000 loading cycles, and long-term durability exceeding 100 days. Intrinsic biodegradability allows safe disposal, mitigating environmental risks of conventional flexible devices. With conformal attachment to the body, the sensor enables real-time monitoring of multiple physiological signals (pulse, breathing, and vocalization) and, when combined with neural-network recognition, achieves precise classification of multiple pulse and laryngeal movements. Moreover, by integrating MASWs into a robotic hand, a human-machine interaction system was established to potentially restore fine motor control in Parkinson’s disease patients, highlighting the sensor’s applicability in intelligent healthcare and IoT interfaces.

## Results

### Design and fabrication of MASW

**Fig. 1:**
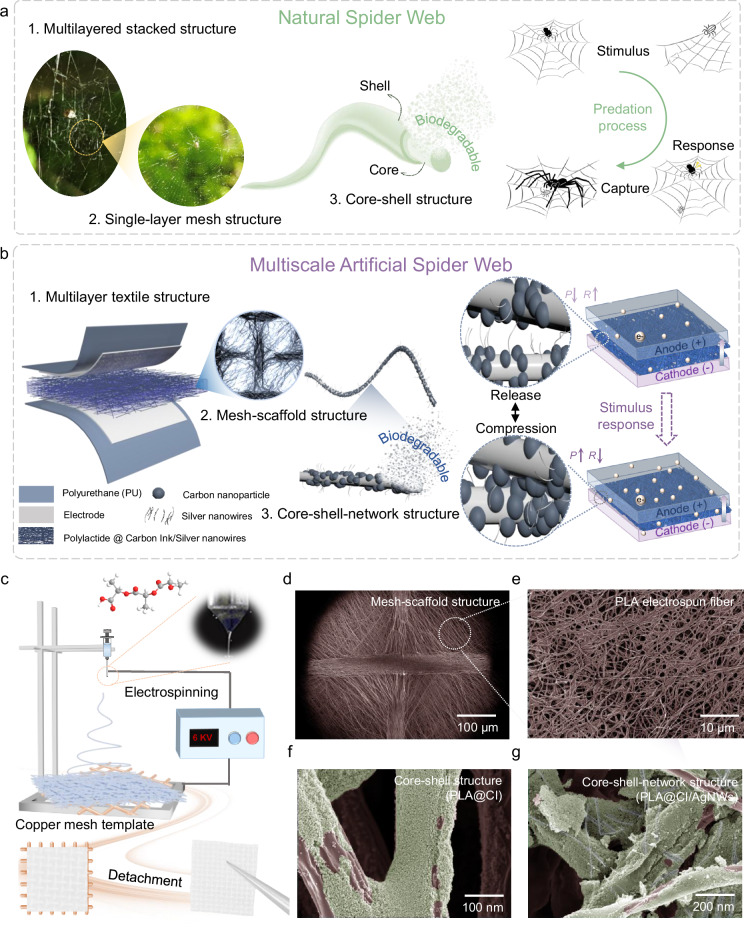
Design, fabrication, and structural characterization of the MASW. **a** Hierarchical structures of the natural spider web and spider predation processes; **b** Hierarchical structures of multiscale artificial spider web and pressure-sensitive mechanisms; **c** Copper-mesh-assisted electrospinning process; SEM images of the multiscale structures of the PLA-based sensing layer, **d** Mesh-scaffold structure (PLA@CI); **e** Electrospun multilayer fiber structure (PLA@CI); **f** Core-shell structure (PLA@CI); **g** Core-shell-network structure (PLA@CI/AgNWs).

Natural spider webs serve as efficient mechanical signal transmission networks, enabling spiders to perceive subtle pressures and vibrations under extremely low energy stimuli during the predation process^12,13^. The multilayered stacked structure, formed by the interlaced accumulation of fibers at different levels, enables efficient stress dispersion and transmission under external forces, thereby ensuring overall structural stability^14^. Meanwhile, the single-layer mesh structure, arranged in regular or semi-regular pores, allows rapid perception and propagation of mechanical stimuli applied from different directions^15^. In addition, the heterogeneous distribution of MaSp1 and MaSp2 proteins across the cross-section of spider silk constitutes a core-shell structure: the shell exhibits a high modulus with low ductility, whereas the core possesses a low modulus with high ductility^16^. Their synergistic effect endows spider web with exceptional strength and toughness (Fig. 1a).

Drawing inspiration from these natural architectures, we designed a MASW that integrates a multiscale conductive three-dimensional (3D) network to achieve high sensitivity, rapid response, and good mechanical stability (Fig. 1b). The MASW was fabricated via a copper mesh-assisted electrospinning, producing polylactic acid (PLA) textiles with a multilayer textile structure (Fig. 1c). This architecture provides continuous pathways for stress dispersion and transmission under external pressure, mitigating local stress concentration and significantly improving both sensitivity and linearity of the sensor.

During electrospinning, a high-voltage electric field stretches the polymer jet and deposits fibers onto the collector^17^. When a copper mesh replaces the conventional flat collector, fibers are preferentially deposited along the conductive lines due to electrostatic guidance, while fewer fibers deposit within the mesh pores. This results in the formation of a hierarchical network skeleton (Fig. 1d)^18^. This mechanism offers a simple and efficient strategy for constructing MASWs. Moreover, the method allows structural tunability: MASWs with different skeleton geometries were fabricated by employing copper meshes with varying sizes (Fig. S1). Such skeletons not only serve as macroscopic frameworks to enhance mechanical stability but also guide ordered stacking and interconnection of fibers at the microscale (Fig. 1e), thereby improving stress transmission. Building on this framework, we further introduced a core-shell-network structure through a dip-coating process: the PLA flexible skeleton was uniformly coated with carbon ink (CI), silver nanowires (AgNWs), or their hybrid conductive networks (CI/AgNWs) (Fig. 1f, g).

The overall resistance of MASW can be expressed as

1 $$R={R}_{{bulk}}+{R}_{{contact}}$$

where R_bulk_ represents the intrinsic resistance of the conductive fillers, and R_contact_ denotes the interfacial contact resistance between conductive units. In this system, the pressure-dependent resistance variation is primarily dominated by changes in contact resistance.

According to Hertzian contact theory^19^, when two elastic bodies are subjected to a normal force *F*, the contact radius *a* can be described as

2 $$a={\left(\frac{3{FR}}{4{E}^{*}}\right)}^{1/3}$$

where R is the equivalent curvature radius and E^*^ is the effective elastic modulus. Since the contact resistance is inversely proportional to the contact area, ${R}_{{contact}}\propto \frac{1}{{a}^{2}}$, the contact resistance decreases with increasing pressure following

3 $${R}_{{contact}}\propto {F}^{-2/3}$$

Consequently, the applied pressure enlarges the effective contact area between conductive units, resulting in a reduction in overall device resistance.

Compared with conventional single-filler systems, the core-shell-network structure in MASW establishes multiscale conductive pathways, including surface contacts (CI shell layers), line contacts (AgNW bridges), and point contacts (carbon particles). The coexistence of these contact modes significantly increases the number of conductive junctions per unit volume and amplifies pressure-induced variations in conductive pathways.

The electrical behavior further follows percolation theory^20^. When the conductive filler fraction φ exceeds the critical percolation threshold ${\varphi }_{c}$, the conductivity follows

4 $$\sigma \propto {(\varphi -{\varphi }_{c})}^{t}$$

During compression, deformation of the framework increases the local filler packing density ($\varphi={\varphi }_{0}+\triangle \varphi (P)$), resulting in a progressive increase in conductivity. The CI/AgNWs hybrid filler effectively lowers the percolation threshold, allowing the conductive network to evolve continuously under pressure.

In addition, nanoscale gaps between conductive particles enable electron transport through tunneling conduction^21^, with the tunneling resistance expressed as

5 $${R}_{t}\propto {e}^{\beta d}$$

where d represents the interparticle distance. External pressure reduces the distance, leading to an exponential decrease in resistance and enabling high sensitivity under small pressure stimuli.

Importantly, the three-dimensional multiscale structure enables progressive contact evolution during compression, where conductive junctions gradually transition from point contacts to line contacts and eventually to surface contacts. This gradual formation of conductive pathways prevents premature conductive saturation and ensures a stable and nearly linear resistance variation over a broad pressure range. Meanwhile, the elastic fiber framework facilitates rapid mechanical deformation and recovery, allowing fast formation and separation of conductive contacts, which enabled highly sensitive detection of subtle pressure and rapid dynamic response on the millisecond timescale. The synergistic integration of multiscale micro/nanostructures combines the flexibility and biodegradability of the PLA skeleton with the superior conductivity of CI/AgNWs, ultimately constructing a biodegradable artificial spider web with high pressure-sensing performance.

### Degradability and breathability of MASW

**Fig. 2:**
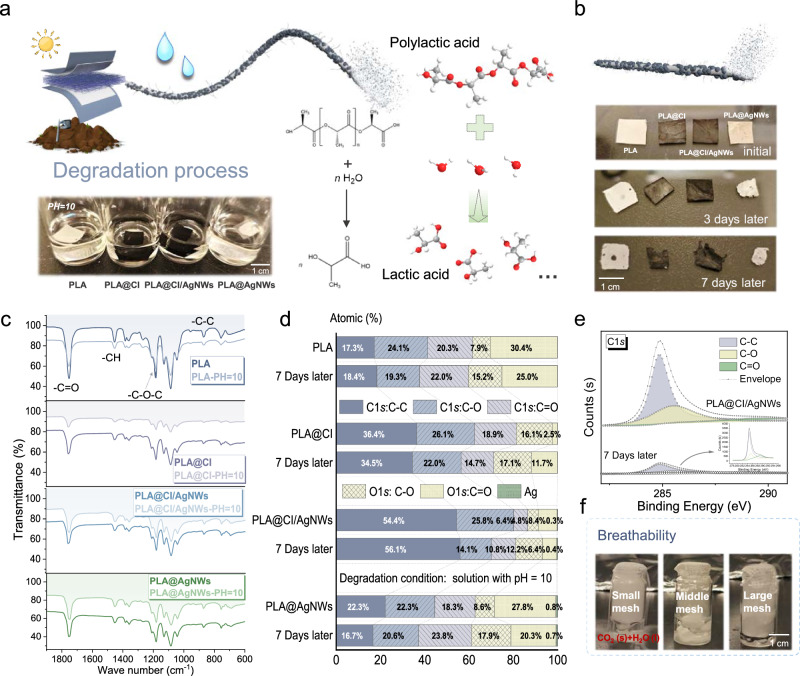
Biodegradability and breathability of MASWs. **a** Schematic illustration of the degradation process of PLA-based pressure-sensitive layers; **b** Optical images of MASWs before and after degradation; **c** FTIR spectra of MASWs before and after degradation; **d** Elemental compositions and chemical bonding states of MASWs before and after degradation based on XPS analysis; **e** XPS peak fitting of carbon in PLA@CI/AgNWs before and after degradation; **f** Breathability verification of the MASWs.

Polylactic acid (PLA) is a thermoplastic aliphatic polyester synthesized from lactic acid monomers, well known for its excellent biocompatibility and biodegradability^22^. Under hydrolysis, the ester bonds in PLA chains gradually cleave to form oligomers and lactic acid molecules (Fig. 2a), ultimately achieving complete degradation into environmentally benign products^23^.

To evaluate the degradability of MASW, we prepared pristine PLA electrospun films (1 cm × 1 cm) and PLA-based composites incorporating three different conductive materials: PLA@CI, PLA@CI/AgNWs, and PLA@AgNWs. These films were immersed in sodium carbonate aqueous solution (pH = 10, simulating alkaline wastewater commonly found in domestic and industrial sources^24^) and maintained at 20 °C. After 7 days, all types of films exhibited pronounced degradation, confirming their favorable environmental degradability (Fig. 2b), which indicates that the MASW can naturally decompose after its service life, preventing long-term accumulation of electronic waste and thereby reducing environmental burdens while promoting the advancement of green wearable electronics.

Further analysis of the films before and after degradation was performed using Fourier-transform infrared spectroscopy (FTIR) (Fig. 2c). The characteristic peaks corresponding to C-C single bonds (~870 cm^−^^1^), C–O–C single bonds (~1083 and ~1181 cm^−1^), and C = O double bonds (~1749 cm^−^^1^) were all attenuated to varying degrees after degradation, indicating cleavage of the main chemical bonds in PLA^25^. X-ray photoelectron spectroscopy (XPS) analysis (Fig. 2d) revealed significant compositional changes in C, O, and Ag elements. After 7 days, the relative content of O increased markedly, reflecting hydrolytic reactions of PLA. In addition, the notable decrease in C content for PLA@CI and PLA@CI/AgNWs films was attributed to the detachment of carbon ink, and the concurrent reduction in C-O bond content further confirms that the PLA matrix underwent degradation into lactic acid molecules. Peak fitting of the C1*s* spectrum of PLA@CI/AgNWs film (Fig. 2e) further demonstrated an increase in C-C content and a decrease in C=O and C-O content after degradation, consistent with the theoretical trend of bond scission during PLA hydrolysis.

Moreover, to intuitively demonstrate the breathability of MASW, we introduced CO₂ generated from the reaction between dry ice and water. The released CO₂ rapidly condensed surrounding water vapor into visible mist-like droplets, serving as tracers for gas diffusion. Experimental results confirmed that these misty droplets permeated quickly and uniformly through MASWs with different mesh-scaffold structures (Fig. 2f, Movie S1), exhibiting efficient breathability, which not only ensures comfort during long-term wear but also supports their practical applicability in wearable electronics.

### Pressure-sensing performance of MASWs

**Fig. 3:**
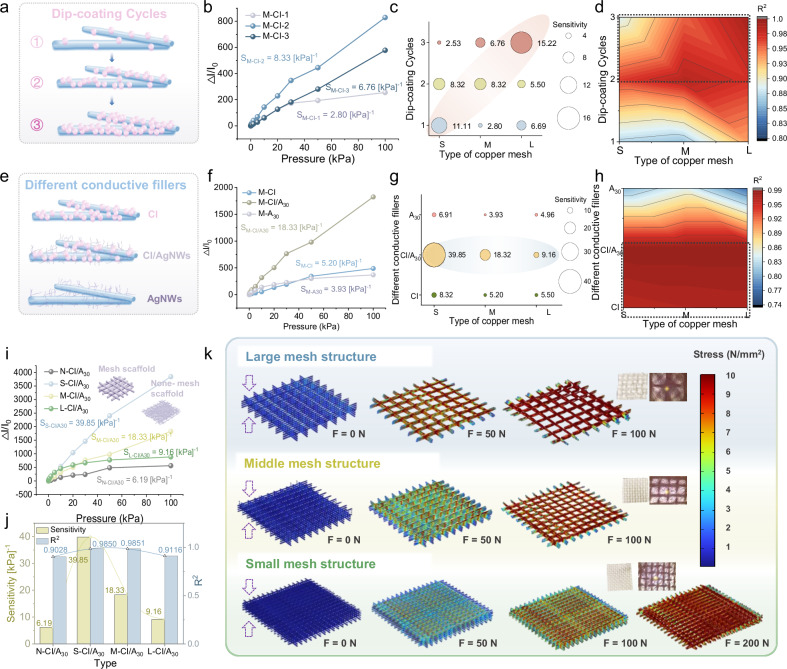
Pressure-sensing performance optimization of MASWs. **a** Schematic illustration of the fabrication of MASWs with different dip-coating cycles; Pressure-sensing performance of MASWs prepared under different dip-coating cycles: **b** Pressure-response curves; **c** Sensitivity; **d** Linearity; **e** Schematic illustration of MASWs fabricated under different conductive fillers; Pressure-sensing performance of MASWs with different conductive fillers: **f** Pressure-response curves; **g** Sensitivity; **h** Linearity; Comparison of pressure-sensing performance between MASWs with different mesh skeleton sizes: **i** Pressure-response curves; **j** Sensitivity and linearity; **k** Finite element simulations of stress distribution in MASWs with different mesh skeleton sizes.

To further investigate and optimize the pressure-sensing performance of MASWs, we first examined the effect of multiple dip-coating cycles of carbon ink on different mesh structures. With increasing dip-coating cycles, the amount of conductive carbon particles adhered to the insulating PLA scaffold gradually increased (Fig. 3a, S2), thereby regulating the effective utilization of the sensing body. MASWs prepared under different conditions all exhibited good pressure response curves (Fig. 3b, S3), while the optimal dip-coating cycle number differed among the various mesh architectures. Specifically, insufficient attachment of conductive particles led to a reduced change in overall conductivity under pressure, resulting in lower sensitivity; conversely, excessive particle loading restricted the conductivity variation range, thereby compromising the average sensitivity across the full pressure range. Because different mesh structures of MASWs present distinct porosities, their corresponding optimal particle loading levels differ accordingly. Consequently, as the mesh size increased, the number of dip-coating cycles required to achieve the optimal sensing-body utilization (corresponding to the maximum sensitivity) also increased (Fig. 3c). Furthermore, statistical analysis indicated that when the number of dip-coating cycles was no less than two, the linearity (R^2^) of the MASWs consistently exceeded 0.95 (Fig. 3d), suggesting that insufficient coating cycles can result in inhomogeneous particle distribution. Therefore, all subsequent studies were carried out with two dip-coating cycles.

Building upon carbon ink as a conductive filler, silver nanowires (AgNWs) were further introduced, with pristine AgNWs serving as a control (Fig. 3e, S4, S5). Notably, MASW using PLA@CI/AgNWs exhibited markedly superior pressure-sensing performance (Fig. 3f, S6), particularly in terms of sensitivity (39.85 [kPa]^−1^) (Fig. 3g), compared with those employing either filler alone. This enhancement can be attributed to the fact that the introduction of AgNWs refined the initial PLA@CI core-shell structure into a core-shell-network configuration (Fig. S4b), constructing an efficient three-dimensional conductive network. This structure greatly facilitated rapid carrier transport and multi-point contact effects, enabling highly sensitive detection of subtle pressures with millisecond-scale dynamic response (42 ms). In contrast to pristine AgNWs, the presence of carbon ink improved the adhesion of the hybrid fillers, ensuring more uniform and stable distribution across the scaffold. This observation is consistent with the fact that MASWs containing carbon ink fillers exhibited better linearity (R^2^ > 0.97) than others (Fig. 3h).

Finally, to elucidate the influence of mesh structures on the pressure-sensing performance of MASWs, three types of MASWs and a non-mesh PLA textile were fabricated, each dip-coated twice with CI/AgNWs. Comparative results show that all MASWs exhibited superior pressure response characteristics compared to the non-mesh PLA textiles (Fig. 3i). This improvement arises from the ability of the mesh scaffold to reinforce the mechanical stability and stress transmission of the fibrous membrane, leading to higher linearity than the non-mesh structure device (Fig. 3j). Moreover, the mesh framework provides effective pathways for stress distribution and dissipation, preventing localized stress concentration and thereby significantly enhancing overall sensitivity. In particular, the small-mesh MASW achieved a 6.44-fold increase in sensitivity relative to the non-mesh PLA textile. Finite element simulations further revealed that all three mesh structures contributed substantially to stress transmission under 0–100 N loading (Fig. 3k, Movie S2). Among them, the small-mesh configuration sustained uniform stress distribution up to 200 N before exhibiting significant deformation, in high agreement with the experimental results.

**Fig. 4:**
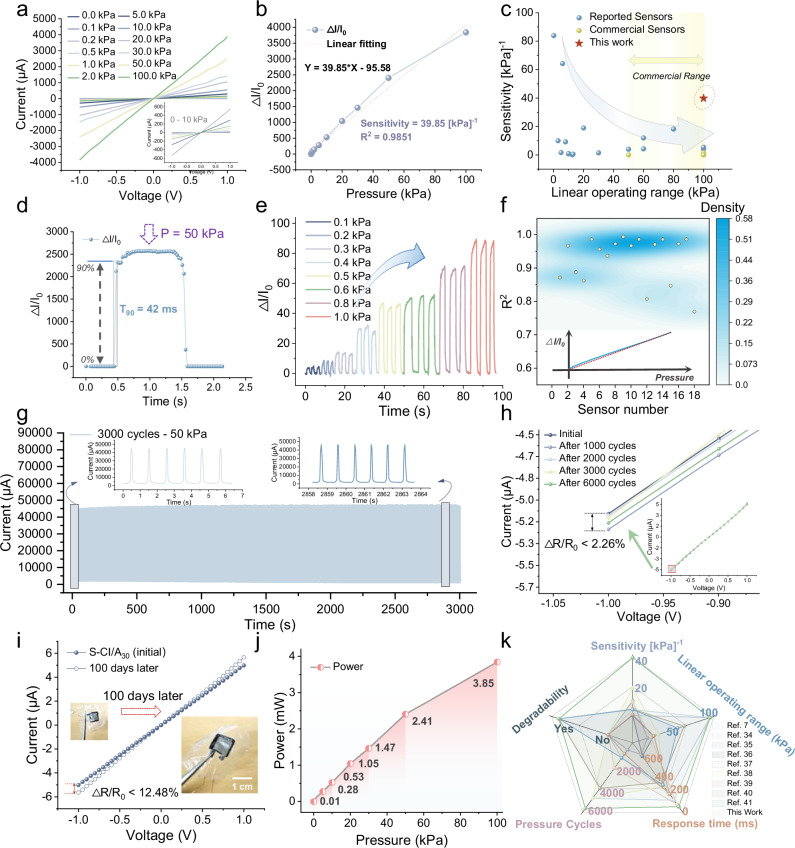
Comprehensive pressure-sensing performance of MASW. **a** I-V characteristics under pressures from 0 to 100 kPa; **b** Pressure response curve and linear fitting; **c** Comparison of sensitivity and linear operating range with reported and commercial flexible pressure sensors; **d** Response time; **e** Dynamic pressure response under small pressures (0.1–1.0 kPa); **f** Statistical analysis of linearity performance of mesh-skeleton-based sensors; **g** Durability under 3000 cycles at 50 kPa; **h** Comparison of initial I-V curves before and after 6000 pressure cycles; **i** Comparison of initial I-V curves before and after 100 days; **j** Power consumption under pressures of 0–100 kPa; **k** Comprehensive performance comparison with reported piezoresistive sensors.

After systematically investigating the effects of dip-coating cycles, conductive filler types, and mesh structures on the pressure-sensing performance of MASWs, we obtained an optimized pressure sensor—MASW with a small mesh skeleton subjected to two dip-coating cycles of CI/AgNWs. This device exhibited stable piezoresistive characteristics (Fig. 4a), achieving an ultrahigh sensitivity of 39.85 [kPa]^−1^ and high linearity (R^2^ = 0.9851) within the pressure range of 0-100 kPa (Fig. 4b). Compared with previously reported and commercial flexible pressure sensors, the MASW exhibited superior performance in both sensitivity and pressure response range (Fig. 4c, Table S1)^7–9,26–44^.

Benefiting from the construction of a 3D conductive network, the applied pressure could be rapidly and uniformly transmitted within the fibrous scaffold, inducing resistance changes and enabling highly sensitive and rapid pressure detection. As a result, MASW exhibited a fast response time (42 ms) (Fig. 4d) and maintained reliable dynamic sensing capability even in the low-pressure range (0–1 kPa) (Fig. 4e).

Moreover, statistical analysis of the pressure-response linearity of 18 devices fabricated with different conditions (Table S2) revealed that 67% of the devices exhibited R^2^ values above 0.9 (Fig. 4f), underscoring the critical role of the mesh skeleton in maintaining structural stability and sensing consistency. MASW also demonstrated robust durability, with no significant changes observed in its microscopic morphology after 6000 loading cycles, exhibiting only a minimal thickness variation of 4.88 μm (Fig. S7). Meanwhile, the resistance variation remained below 2.26% (Fig. S8, 4g, h) further confirming its reliable mechanical stability and electrical reliability. Furthermore, after 100 days of natural environmental exposure, the I-V measurements showed only a 12.48% variation in initial resistance (Fig. 4i), confirming the long-term durability of MASW.

Notably, the maximum power consumption of MASW was only 3.85 mW under its full-scale working pressure (Fig. 4j), which not only minimizes energy consumption but also provides the potential for large-scale integration into wearable electronic devices. Finally, a systematic comparison with previously reported resistive flexible pressure sensors, which were evaluated in terms of sensitivity, linear operating range, response time, pressure cycles, and degradability, demonstrated that MASW exhibits overall superior performance (Fig. 4k, Table S3)^7,37–44^, highlighting the tremendous potential of MASW as a next-generation green flexible pressure sensor.

### Application of MASW in physiological signal monitoring and recognition

**Fig. 5:**
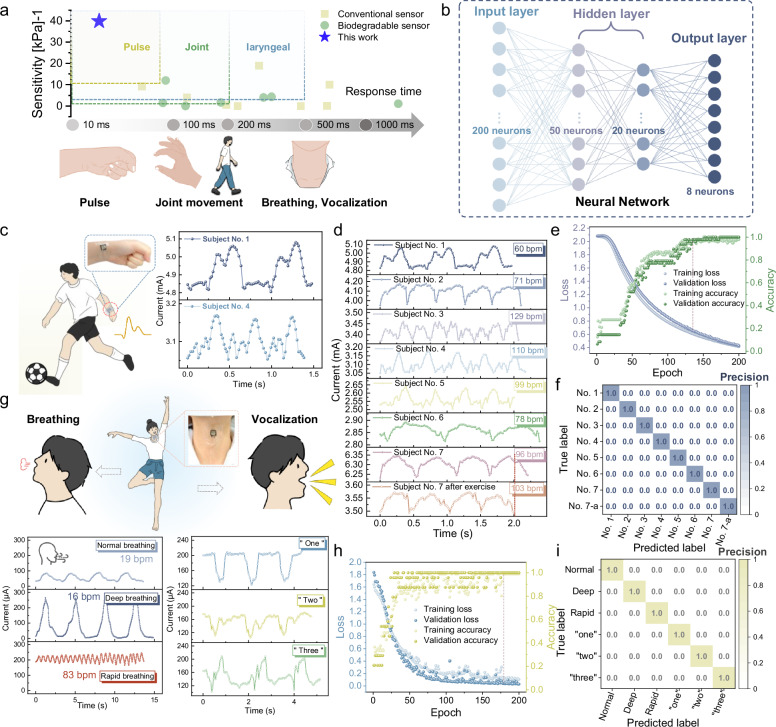
Advanced physiological signal monitoring and assessment enabled by MASW. **a** Monitoring requirements of advanced physiological signals (response time and sensitivity); **b** The components of neural network; **c** Schematic illustration of pulse monitoring via MASW; **d** Pulse signals from different individuals (intensity, frequency, waveform); **e** Loss and accuracy in classifying eight different pulse signals; **f** Confusion matrix for classification of eight different pulse signals; **g** Schematic illustration of breathing and vocalization monitoring via MASW; **h** Loss and accuracy in classifying six different laryngeal movement signals; **i** Confusion matrix for classification of six different laryngeal movement signals.

During physiological activities, the human body generates diverse pressure signals, such as pulse, joint movement, breathing, and vocalization. These signals not only reflect the fundamental states of the cardiovascular, respiratory, and motor systems but also serve as important indicators for early disease screening and health monitoring^45^. Therefore, real-time and accurate monitoring of these pressure signals holds significant scientific and practical value. Compared with previously reported conventional and biodegradable pressure sensors, MASW, with its combination of ultrahigh sensitivity (39.85 [kPa]^−1^) and rapid response (42 ms), effectively meets the requirements of detection ranges for monitoring all the major physiological signals (Fig. 5a, Table S4)^8,9,31–44^.

Leveraging the reliable sensing capabilities of MASW, we further developed a neural network using the Transformer model for intelligent processing of the acquired signals and precise evaluation of human physiological states (Fig. 5b)^46^. The neural network is trained by inputting large volumes of pressure signal data, after which it can accurately recognize and classify different pressure signals. The Transformer model captures long-range dependencies in time-series pressure signals via self-attention, enabling accurate extraction of subtle temporal patterns that are difficult to resolve using conventional convolutional or recurrent approaches.

Pulse signals are closely related to physical activity, health status, and often serve as critical indicators for disease warning. For instance, post-exercise acceleration of pulse reflects exercise intensity, while irregular pulses may indicate arrhythmia^47^. When MASW was worn on the wrist (Fig. 5c), we collected pulse signals from seven subjects at rest and additionally recorded post-exercise pulse signals from one subject (Fig. 5d, S9). Each group of signals was repeatedly gathered 300 times, with 80% used for neural network training and 20% for validation. After 130 epochs, the model achieved a loss of less than 0.613 and a classification accuracy of 1 (Fig. 5e). Furthermore, it demonstrated 100% precision in distinguishing eight different pulse signals (Fig. 5f), effectively capturing differences in intensity, frequency, and waveform, confirming its reliable capability in fine-grained physiological signal monitoring and assessment.

Throat signals reflect airway conditions, phonation patterns, and are even associated with clinical diagnostics such as sleep apnea and neurological disorders^48^. Similarly, MASW was attached to the throat for real-time monitoring of laryngeal movements during breathing and vocalization (Fig. 5g). In this case, after 170 epochs, the neural network stabilized with a loss of less than 0.054 and a recognition accuracy of 1 (Fig. 5h). Ultimately, the model achieved 100% precision in identifying six different laryngeal movement states (Fig. 5i), demonstrating the great potential of MASW in complex physiological signal monitoring and health state assessment.

### Applications of MASW in human-machine interaction

**Fig. 6:**
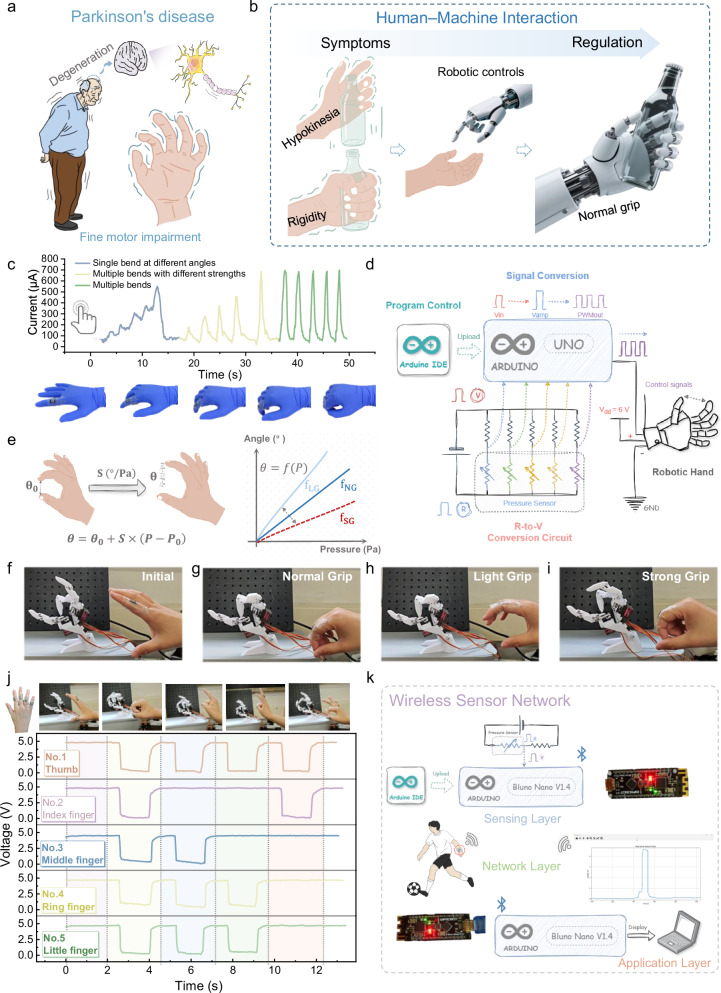
Human-machine interaction enabled by MASW. **a** Illustration of Parkinson’s disease symptoms; **b** Correction strategies via human-machine interaction; **c** Monitoring of finger movements with varying angles, forces, and repetitions; **d** Circuit diagram for MASW-controlled robotic hand; **e** Adjustable robotic hand control using sensitivity factors; MASW-controlled robotic hand under different states: **f** Initial state; **g** Normal grip state; **h** Light grip state; **i** Strong grip state; **j** Complex gesture control of the robotic hand; **k** Schematic diagram of the wireless sensor network.

According to the World Health Organization (WHO), Parkinson’s disease (PD) affects approximately 8.5 million people worldwide, particularly among the elderly population^49^. The typical pathological features of PD include degeneration and loss of dopaminergic neurons, dopamine deficiency, and neurotransmitter imbalance, which collectively impair fine motor function during normal physiological activities (Fig. 6a)^50,51^. Among the most representative clinical symptoms of PD is finger dexterity impairment. For example, hypokinesia manifests as reduced movement amplitude and slower response speed, while grip rigidity results in excessive grip force. Both conditions significantly compromise fine motor control of the hands^52^.

To address these challenges, we applied MASW in the field of human-machine interaction by collecting pressure signals generated during gripping and establishing a control circuit for robotic hand, enabling patients in different states to perform normal grasping (Fig. 6b). Benefiting from its high sensitivity, MASW can accurately monitor finger movements at different angles, forces, and repetitions, thereby providing precise input signals for human-machine interaction system (Fig. 6c). On this basis, we designed and constructed a resistance-voltage (R-V) conversion circuit that converts resistance variations of MASW under pressure into voltage signals through a voltage divider, which are then transmitted to an Arduino UNO board. The board, via programmed instructions, converts these voltage signals into PWM signals with different duty cycles and outputs them to the robotic hand’s control circuit, enabling diverse motions under the combined action of working and control voltages (Fig. 6d).

Initially, a single sensor was attached to one finger to control the robotic hand, enabling adjustable grip force. As shown in Fig. 6e, the joint angle θ of the robotic hand is determined by finger pressure P and a sensitivity factor S, following the relationship:

6 $$\theta={\theta }_{0}+S\times \left(P-{P}_{0}\right)$$

where P₀ is the baseline pressure obtained during calibration and θ₀ is the initial angle of the robotic hand. Compared with the angle-pressure curve of normal grip (Fig. 6f, g), light grip exhibits a steeper slope, indicating that less pressure is required for the robotic hand to achieve the same angular motion (Fig. 6h), consistent with the motor state of patients with hypokinesia. In contrast, strong grip shows a gentler slope, requiring greater pressure for the same angular displacement (Fig. 6i), which aligns with the clinical manifestation of grip rigidity (Movie S3). The slope of the angle-pressure curve in this human-machine interaction system can be readily tuned by modifying the Arduino UNO program, enabling easy customization for individual users and demonstrating significant potential in the field of personalized prosthetics and rehabilitation.

Furthermore, by attaching five sensors to the five fingers, we achieved complex gesture control of the robotic hand (Fig. 6j, Movie S4). The robotic hand successfully reproduced a wide range of complex human gestures, including speed-variable grasping motions, diverse finger arrangements, and symbolic gestures such as “OK,” “Yeah,” and “Great”. This multi-channel input provides a closer approximation of natural hand movements, highlighting its potential applications in medical rehabilitation, intelligent prosthetics, virtual reality, and broader human-machine interfaces.

Finally, we established a wireless sensor network using Arduino Bluno Nano V1.4 for remote physiological signal monitoring (Fig. 6k). The sensing layer can capture resistance signals from MASW in real time and transmit them wirelessly via Bluetooth within a range of 10 meters. The display layer then receives these signals and presents them on a Python-based visualization interface in real time, thereby overcoming spatial limitations and enabling intuitive, remote physiological monitoring. This design significantly broadens the application landscape of MASW, enabling its integration into future intelligent healthcare systems, wearable electronics, and advanced human-machine interaction technologies.

## Discussion

This study presents an artificial spider web that overcomes the performance trade-off in conventional pressure sensors through a multiscale core-shell-network architecture with a 3D fiber skeleton inspired by natural spider webs. The MASW transduces external pressure into electrical signals via its three-dimensional interconnected network, where efficient stress transmission and multidimensional conductive pathways ensure both high sensitivity and rapid response. Simultaneously achieving ultrahigh sensitivity (39.85 [kPa]^−1^), fast response (42 ms), and remarkable durability (6000 cycles, 100 days), the MASW fully covers the detection ranges of all major physiological signals. This capability allows real-time monitoring of diverse physiological signals, including pulse, breathing, vocalization, and joint movements. Such a combination of performance metrics is rarely achieved in previously reported flexible pressure sensors.

Assisted by a transformer-based neural network, it achieves precise recognition and classification of these physiological signals while maintaining eco-friendly biodegradability. Furthermore, the MASW-based adaptive robotic hand control system (from adjustable grasping to complex gesture recognition) demonstrates promising potential for assisting fine motor rehabilitation in patients with Parkinson’s disease. In addition, a wireless sensor network enables remote signal transmission and real-time visualization, overcoming the spatial constraints of traditional wired systems. Collectively, this work highlights MASW as a promising route toward sustainable flexible electronics for smart healthcare, personalized prosthetics, and next-generation human-machine interfaces.

## Methods

### Materials

Polylactic acid (PLA, 4032D) was purchased from NatureWorks (USA). Dichloromethane (DCM, ≥99.5%) and N, N-dimethylformamide (DMF, ≥99.5%) were purchased from Sigma-Aldrich. Carbon ink (particle size ~60 nm) was supplied by Platinum Pen Co., Ltd. (Japan), and silver nanowires (AgNWs, length ~30 μm) were purchased from Novarials. Graphite conductive adhesive (A528) was obtained from Xinwei Electronic Materials Co., Ltd. (China), and plain conductive fabric was purchased from Qingdao Shir Textile Co., Ltd. (China). Deionized (DI) water was purchased from Samchun Chemical (Korea), and isopropyl alcohol (IPA) was purchased from Fisher Scientific (USA).

### Fabrication of MASW

#### Fabrication of electrospun fiber membranes

2.4 g PLA granules were dissolved in 20 mL of a DCM/DMF mixture (7:3 v/v) and stirred in a water bath at 40 °C for 2 h to obtain a uniform spinning solution (10 wt%). A 10 mL syringe with a 21 G needle was used for electrospinning under environmental conditions of 35 °C and 55% RH. The electrospinning parameters were as follows: receiving distance, 12 cm; feeding rate, 0.8 μL/min; applied voltage, 6.0 kV. Copper meshes with mesh sizes of 10, 20, and 30 (10 × 10 cm) were placed on the collector. After 2 h of electrospinning, PLA nanofiber films with embedded mesh-like skeletons were obtained. The films were dried at 50 °C for 6 h, carefully peeled off from the copper mesh, and cut into 1.0 × 1.0 cm sheets for subsequent use.

#### Fabrication of pressure-sensitive layers

A 10 wt% carbon ink/ethanol solution and a 0.1 wt% AgNWs/IPA solution were prepared and mixed at a mass ratio of 10:1, followed by stirring at 30 °C for 10 min to obtain a homogeneous carbon ink/AgNWs solution. The PLA textile films were immersed in solutions containing carbon nanoparticles, AgNWs, or their mixture for 2 min, dried at 50 °C for 2 h, and yielded uniform conductive black films with core-shell/core-shell-network architectures. Repeated dip-coating cycles were performed to regulate the utilization of the sensitive material. (Fig. S10).

#### Assembly of sensors

Plain conductive fabric was cut into 0.8 × 0.8 cm pieces and used as flexible electrodes. Copper wires were fixed at one corner of the electrodes with graphite conductive adhesive and cured at 40 °C for 2 h. The electrodes were laminated above and below the pressure-sensitive layer, and the entire structure was encapsulated with polyurethane (PU) to form a sandwich-type device.

### Characterization and performance testing of the MASW

The surface morphologies of the samples were characterized using field-emission scanning electron microscopy (FESEM, ZEISS, Germany). The X-Ray Photoelectron Spectroscope (XPS) was measured using AXIS-His (KRATOS). The Fourier Transform Infrared (FTIR) was measured using Nicolet iS50 (Thermo Fisher Scientific). The Energy Dispersive Spectroscopy (EDS) was measured using Field-Emission Scanning Electronic Microscopy AURIGA (Carl Zeiss). The surface topology and phase were examined with an atomic force microscope (AFM, NX-10, Park Systems). The electrical properties of the devices were measured with a semiconductor parameter analyzer (Keithley B1500A, Keysight). During dynamic response measurements, a constant bias voltage of 1 V was applied across the sensor, and the corresponding output current was recorded in real time. The participants provided their informed consent to participate in this study, and informed signed consent was obtained from the volunteer.

### Finite element simulation

The model was meshed with triangular elements (Fig. S11). During the simulation, different pressures were applied to the upper and lower boundaries, while the remaining boundaries were left free. Stress distribution of multilayer mesh structures (1.0 × 1.0 cm, with varying mesh sizes) was simulated under applied pressure ranging from 0 to 200 N.

### Parameter definition

The sensitivity of the sensor reported in this work refers to the normalized sensitivity, defined as:

7 $$S=\frac{\Delta I/{I}_{0}}{\Delta P}$$

Where △I is the relative current variation, ${I}_{0}$ is the initial current without applied pressure, and △P is the applied pressure variation. The sensitivity is not constant over the entire pressure range due to the nonlinear contact evolution and conductive pathway reconstruction under compression. In this work, the reported sensitivity represents the average sensitivity within the 0–100 kPa pressure range, calculated from the slope of the best linear fitting of the corresponding pressure-response curve.

The linearity of the sensor was evaluated by fitting the measured pressure-response curve using the least-squares method. The coefficient of determination (R^2^) of the linear fit was calculated to quantify the linearity.

The response time was defined as the time required for the output signal to increase from 0% to 90% of its steady-state value (T_90_).

### Neural network model

A neural network model using the Transformer architecture was employed. The Transformer relies entirely on self-attention mechanisms to model input and output waveform representations, without using sequence-aligned recurrent neural networks (RNNs) or convolutional operations. This design enables efficient modeling of long-range dependencies while allowing parallel computation during training, thereby improving computational efficiency.

Raw signals were first interpolated to ensure a uniform number of data points across different samples. Subsequently, dimensionality reduction was performed using an autoencoder to extract compact latent representations and reduce redundancy in the original feature space. The compressed features were then fed into a softmax classifier for pressure signal recognition and movement state identification.

For pulse signal monitoring and recognition, the classifier consisted of one input layer (200 neurons), two hidden layers (50 and 20 neurons), and one output layer (8 neurons). Eight distinct pulse categories were included, with 300 samples collected per category, resulting in a total of 2400 samples.

For laryngeal movement monitoring and recognition, the classifier included one input layer (100 neurons), two hidden layers (50 and 20 neurons), and one output layer (6 neurons). Six different laryngeal movement categories were involved, with 300 samples per category, yielding a total of 1800 samples.

The activation function used in all hidden layers was the Rectified Linear Unit (ReLU), while the output layer employed the softmax activation function to generate probability distributions over the target classes.

The dataset was randomly divided using stratified sampling, with 80% of the data used for training (cross-validation) and 20% reserved for independent testing. For pulse recognition, this corresponds to 1920 training samples and 480 testing samples. For laryngeal movement recognition, 1440 samples were used for training and 360 for testing.

The model was trained using the Adam optimizer with an initial learning rate of 1 × 10^−3^. The batch size was set to 32, and the maximum number of training epochs was 300. Categorical cross-entropy was adopted as the loss function. Early stopping (patience = 30 epochs) was applied to prevent overfitting, and the learning rate was adaptively reduced using a ReduceLROnPlateau strategy (reduction factor = 0.5, minimum learning rate = 1 × 10^−6^).

To further mitigate overfitting due to the relatively moderate dataset size, dropout regularization (rate = 0.3–0.4) and batch normalization were incorporated into the network.

All experiments were conducted with a fixed random seed to ensure reproducibility.

### Application system design

#### Human-machine interaction system

The system comprised an R-V conversion circuit, a program control module, and a robotic hand. The overall system was using an Arduino UNO microcontroller with Arduino IDE as the development platform. The R-V conversion circuit collected resistance signals from the pressure sensor and converted them into voltage signals, which were fed into the microcontroller. The programmed controller then transformed the input voltage signals into PWM signals, whose duty cycles determined the joint movement angles of the robotic hand. The homemade robotic hand was powered by an external 6 V source.

#### Wireless sensor system

The system consisted of a signal acquisition module, a pressure sensor, and a wireless display module. An Arduino Bluno Nano V1.4 microcontroller with a built-in Bluetooth module was used for wireless communication. The signal acquisition module incorporated a biasing circuit to collect resistance signals from the pressure sensor, convert them into voltage signals, and transmit them via Bluetooth to the display module. Data visualization was achieved through a Python-coded interface.

## Supplementary information

Supplementary Information

Description of Additional Supplementary Files

Movie S1

Movie S2

Movie S3

Movie S4

Transparent Peer Review file

## Source data

Source Data

## Data Availability

The authors declare that the main data supporting the findings of this study are available within the article and its Supplementary Information files. Source Data are provided with this paper. All data are available from the corresponding author upon request Source data are provided with this paper.
