# Supplementary material for: Multiscale artificial spider web for comprehensive pressure sensing and human-machine interaction
Source: Nat Commun. 2026 Jul 4;17:8316. doi: 10.1038/s41467-026-74200-y (PMC13470477; doi:10.1038/s41467-026-74200-y)
Supplement: Supplementary file 1 [file 41467_2026_74200_MOESM1_ESM.pdf]

Supplementary information for

Multiscale Artificial Spider Web for Comprehensive Pressure Sensing and Human-Machine Interaction

*Jing Dai[1,2†], Kwan-Nyeong Kim[1†], Guangzhong Xie[2], Dong-Su Choi[1], Seung-Woo Lee[1], Eojin Yoon[1], Chan-Yul Park[1], Han Dai[3], Somin Kim[1], Hyun-Haeng Lee[1], and Tae-Woo Lee[1,4]**

[1]Department of Materials Science and Engineering, Seoul National University, Seoul, Republic of Korea.

[2]School of Optoelectronic Science and Engineering, University of Electronic Science and Technology of China, Chengdu, P. R. China

[3]Institute of Art and Art Design, Nanyang Normal University, Nanyang, P. R. China

[4]Institute of Engineering Research, Soft Foundry, Research Institute of Advanced Materials, Interdisciplinary Program in Bioengineering, Seoul National University, Seoul, Republic of Korea

*Corresponding author: Tae-Woo Lee, twlees@snu.ac.kr

†These authors contributed equally: Jing Dai, Kwan-Nyeong Kim

The PDF file includes:

- Fig. S1 to S11
- Tables S1 to S4
- References

Table of Contents

Fig. S1. Optical photographs of copper meshes with different mesh sizes and the corresponding MASWs

Fig. S2. EDS characterization of MASWs prepared by different dip-coating cycles

Fig. S3. Pressure–response curves of MASWs after various dipping cycles

Fig. S4. EDS characterization (C, O, and Ag elements) of MASWs using different conductive fillers

Fig. S5. CAFM characterization of MASWs with different conductive fillers

Fig. S6. Pressure–response curves of MASWs using various conductive fillers

Fig. S7. SEM characterization of MASW at different scales before and after 6000 cycles of loading pressure.

Fig. S8. Durability test of the MASW under 3000 loading cycles at 5 kPa

Fig. S9. Pulse signals collected from different individuals

Fig. S10. Schematic illustration of the fabrication process of MASW by dip-coating method

Fig. S11. Finite element simulation mesh division of different mesh structures

Table S1. Comparison of sensitivity and linearity of reported and commercial flexible pressure sensors

Table S2. Summary of the linearity of pressure–response curves for MASWs

Table S3. Overall performance comparison of flexible pressure sensors

Table S4. Comparison of sensitivity and response time of conventional and biodegradable sensors

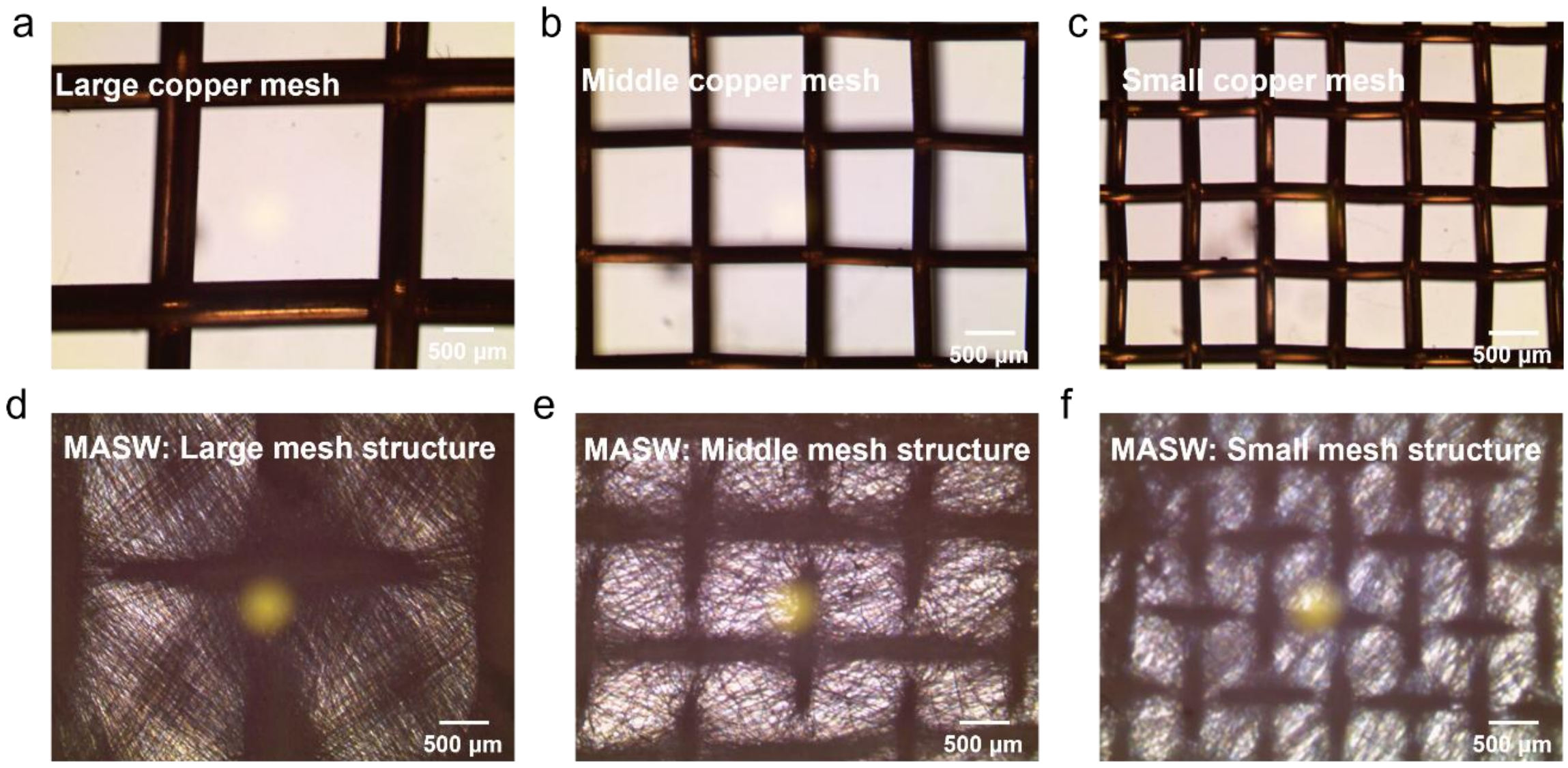

Figure S1. Optical photographs of copper meshes with different mesh sizes and the correspo nding MASWs. (a) Large copper mesh (10-mesh); (b) Middle copper mesh (20-mesh); (c) Small copper mesh (30-mesh); (d) MASW with large mesh structure; (e) MASW with middle mesh structure; (f) MA SW with small mesh structure.

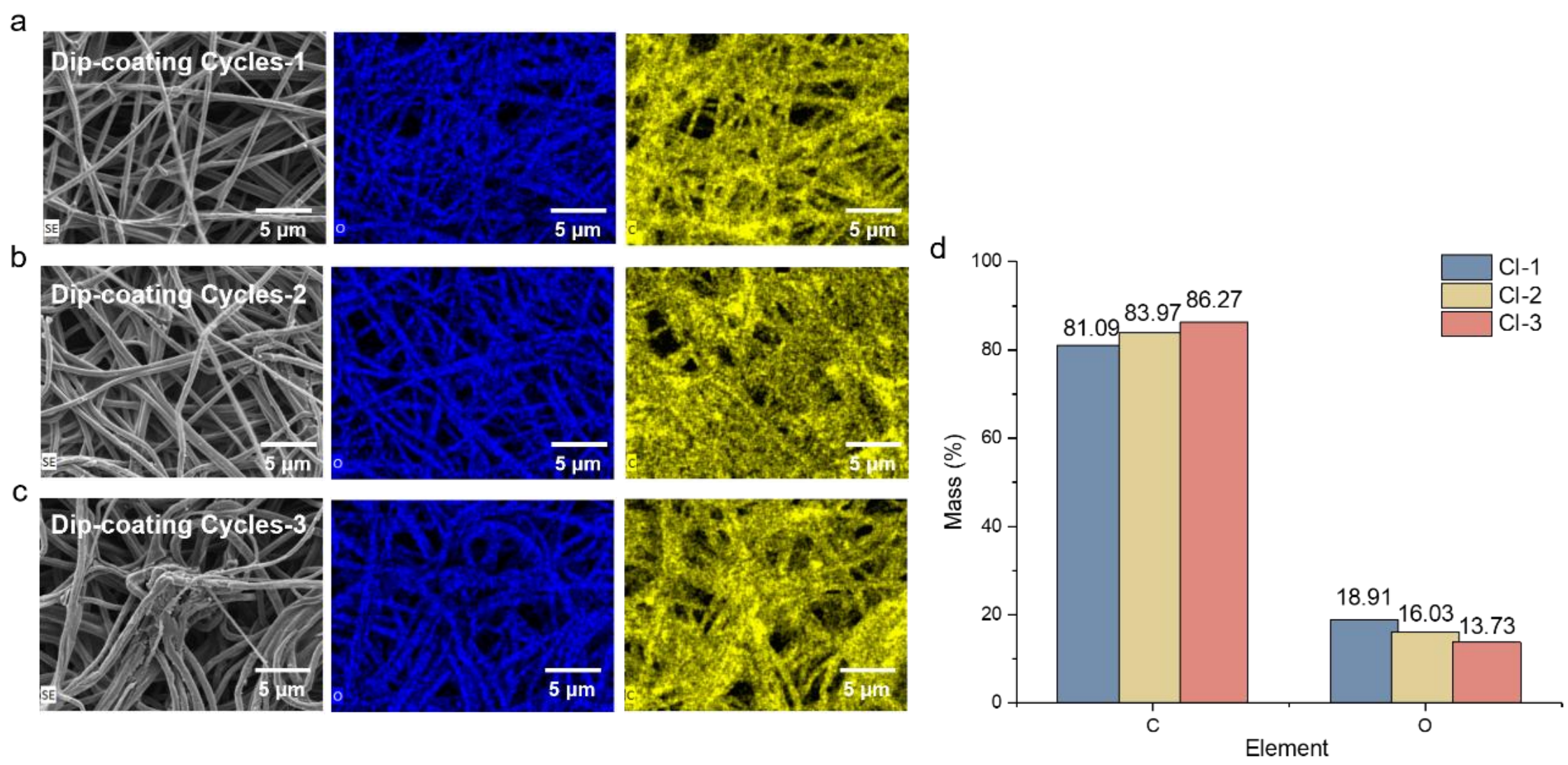

Figure S2. Energy Dispersive Spectrometer (EDS) characterization (C and O elements) of MASWs prepared by different dip-coating cycles in carbon ink. (a) One dip-coating cycle; (b) Two dip-coating cycles; (c) Three dip-coating cycles; (d) Statistical analysis of C and O element contents in MASWs after different dip-coating cycles.

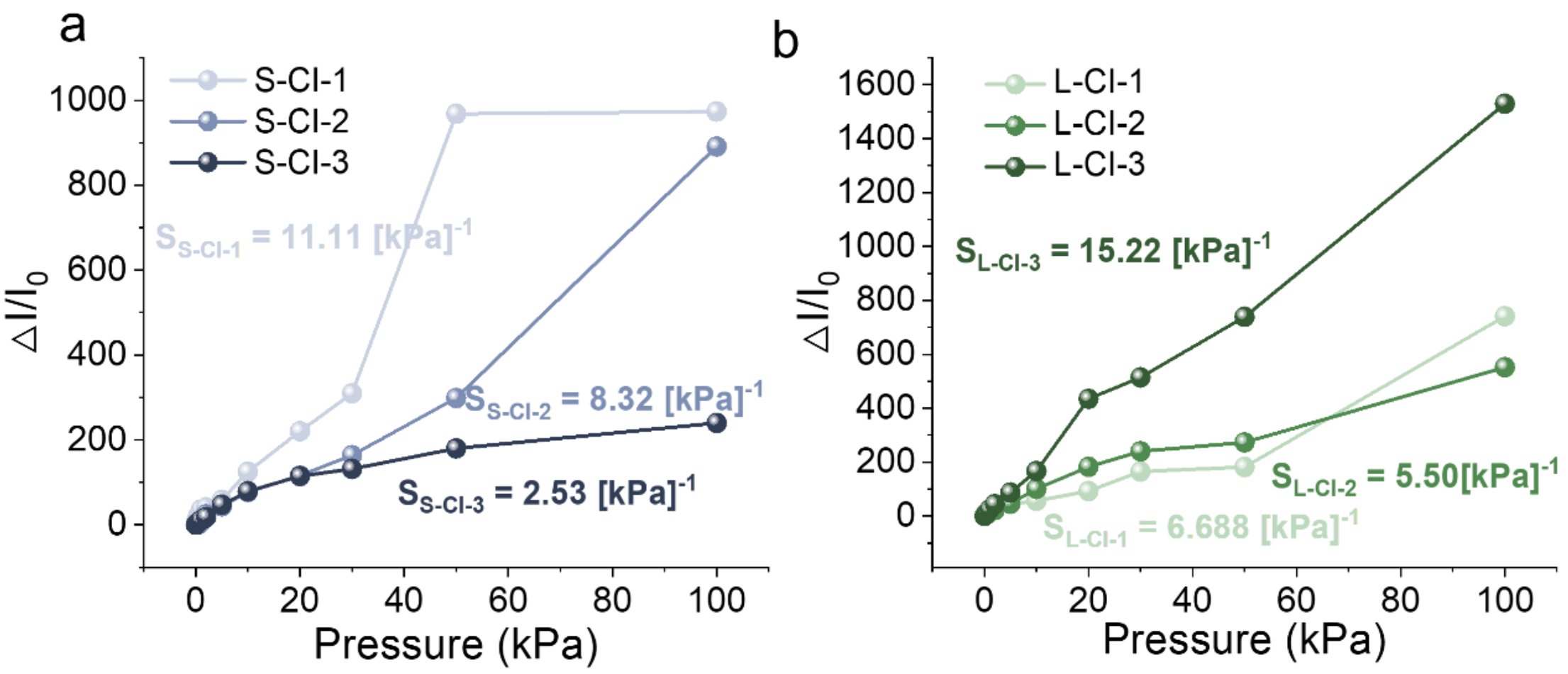

Figure S3. Pressure–response curves of MASWs with different mesh structures after various dipping cycles in carbon ink. (a) Small mesh structure; (b) Large mesh structure.

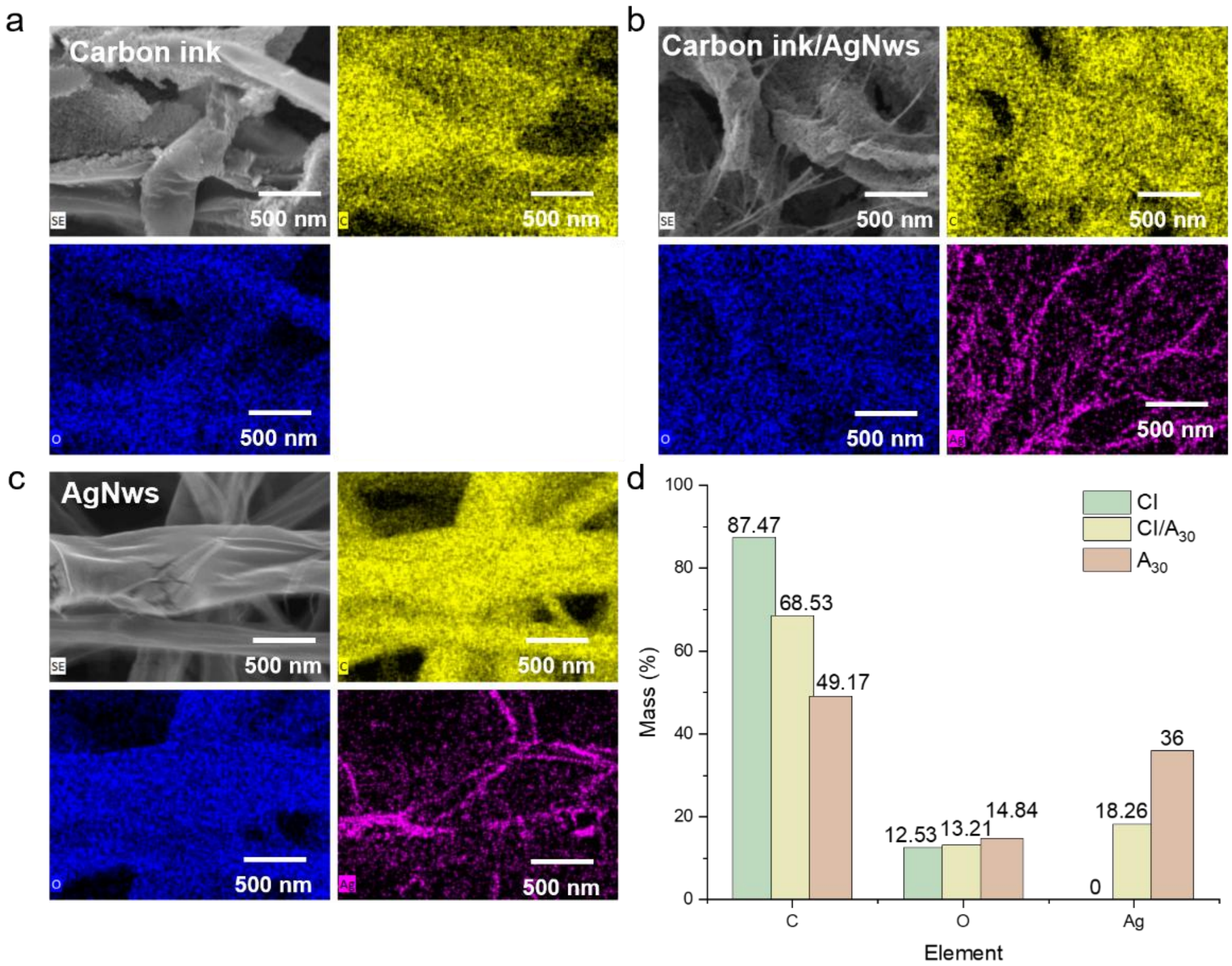

Figure S4. Energy Dispersive Spectrometer (EDS) characterization (C, O, and Ag elements) of MASWs using different conductive fillers. (a) Pure carbon ink; (b) Carbon ink mixed with AgNWs; (c) Pure AgNWs; (d) Statistical analysis of C, O, and Ag element contents in MASWs prepared with different conductive fillers.

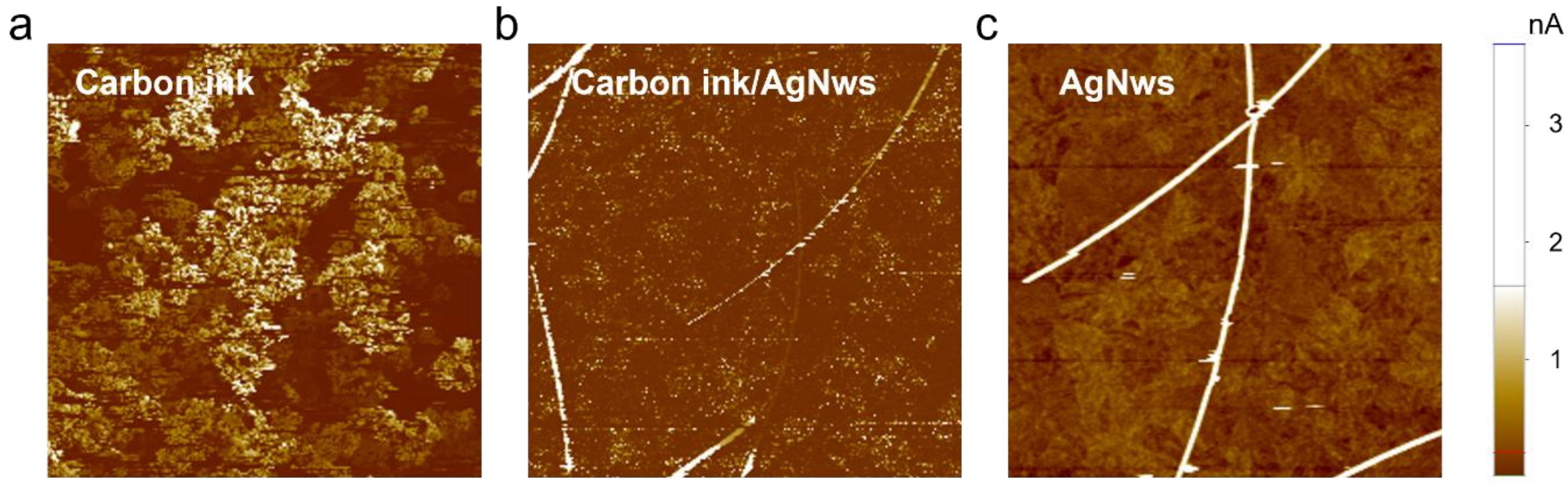

Figure S5. Conductive atomic force microscopy (CAFM) characterization of MASWs with different conductive fillers. (a) Pure carbon ink; (b) Carbon ink mixed with AgNWs; (c) Pure AgNWs.

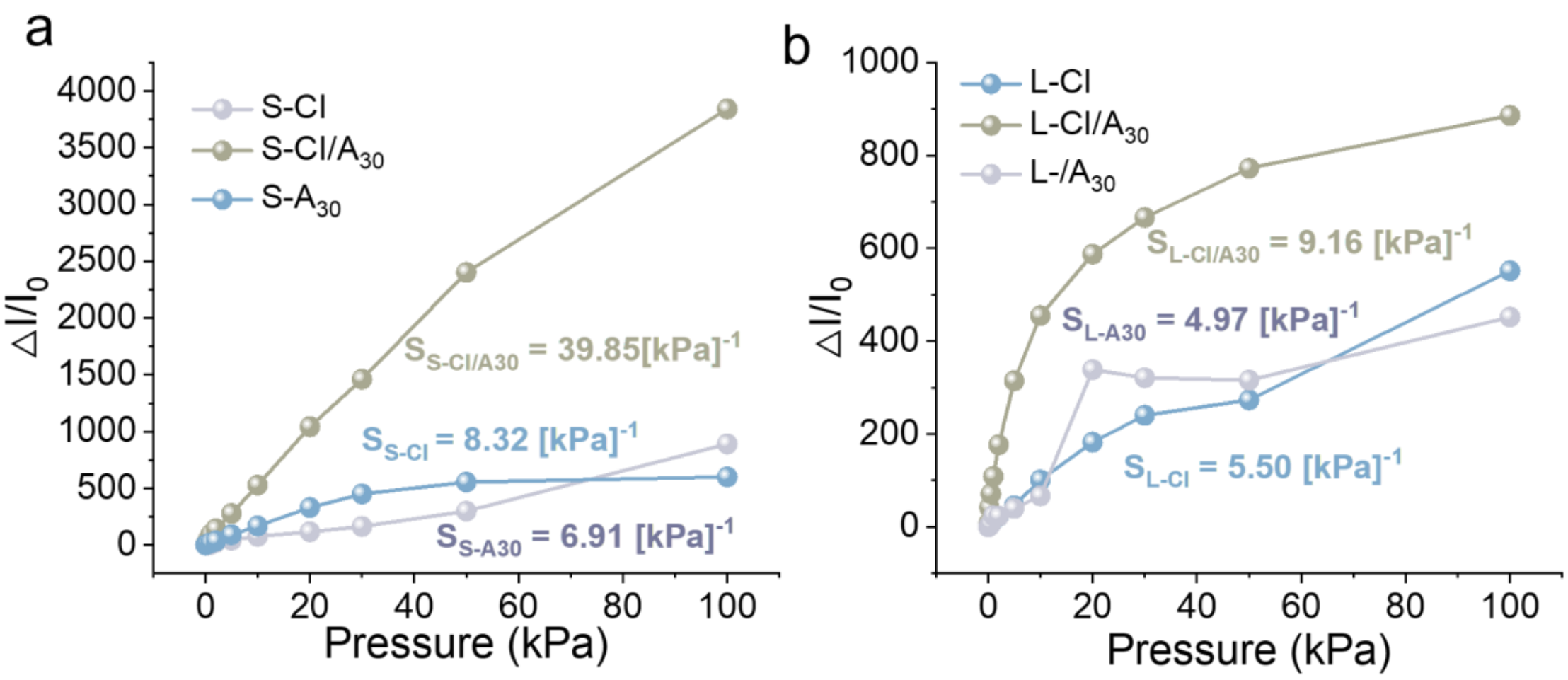

Figure S6. Pressure–response curves of MASWs with different mesh structures using various conductive fillers. (a) Small mesh structure; (b) Large mesh structure.

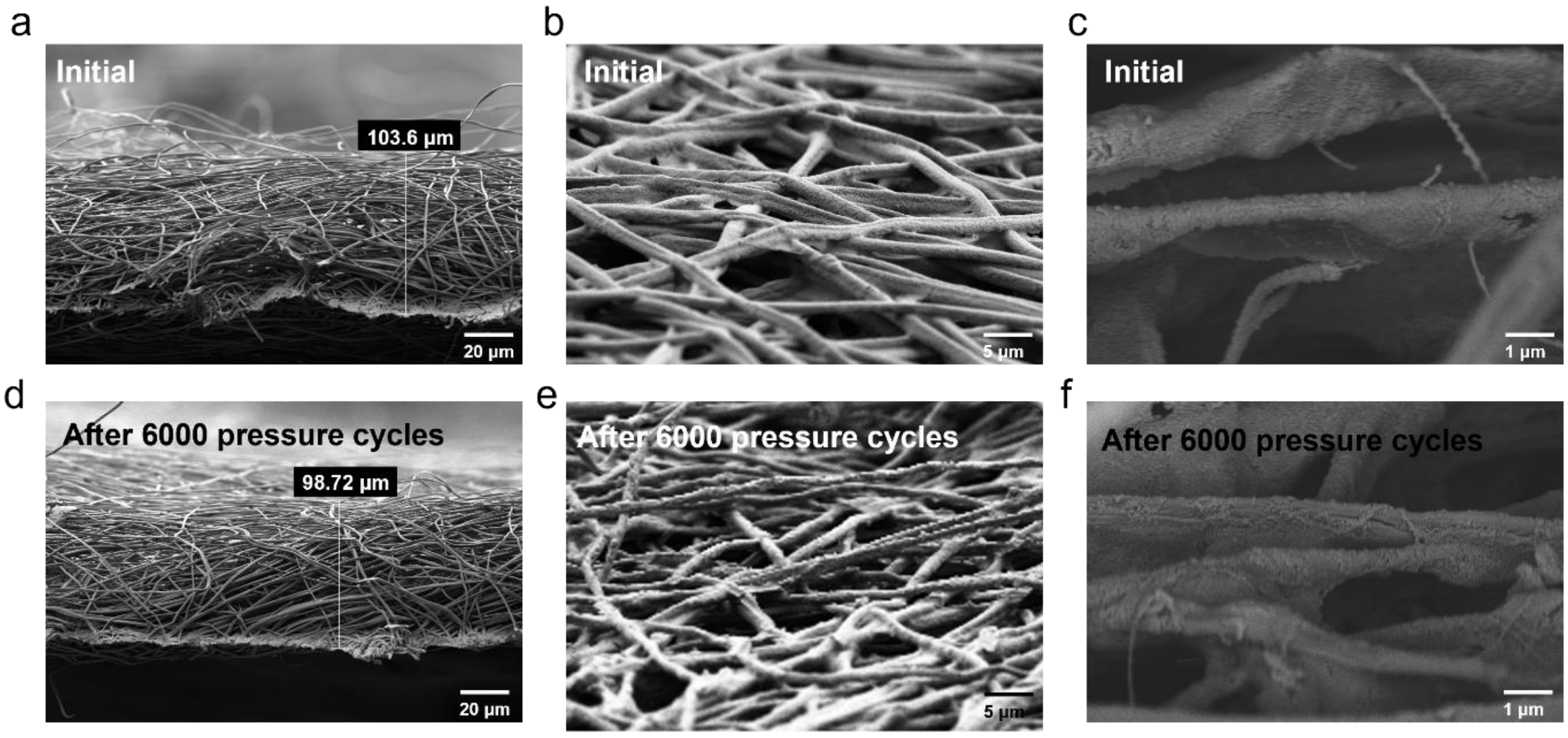

Figure S7. Cross-sectional SEM characterization of MASW at different scales and states. (a) Initial state at 20 μm scale; (b) Initial state at 5 μm scale; (c) Initial state at 1 μm scale; (d) After 6000 loading pressure cycles at 20 μm scale; (e) After 6000 loading pressure cycles at 5 μm scale; (f) After 6000 loading pressure cycles at 1 μm scale.

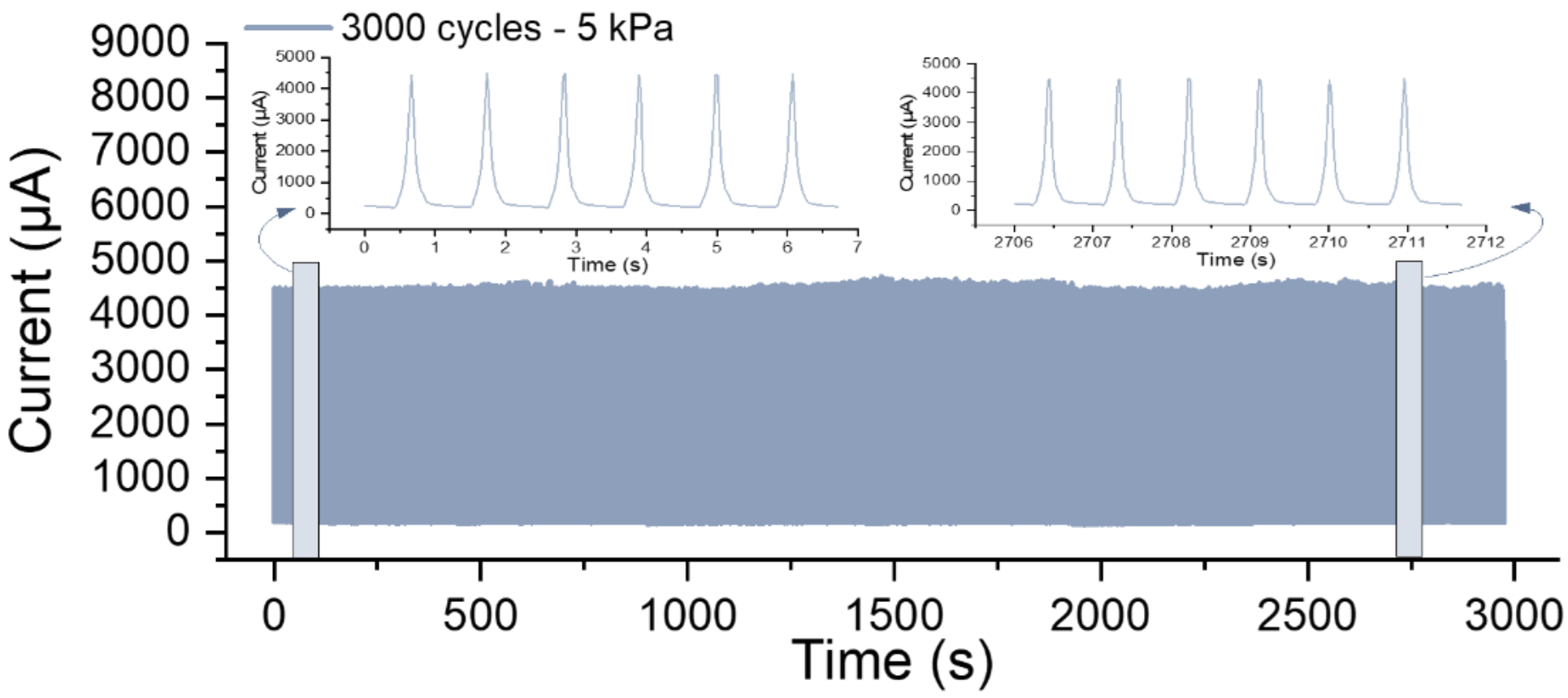

Figure S8. Durability test of the PLA@CI–AgNWs MASW using small mesh structure under 3000 loading cycles at 5 kPa.

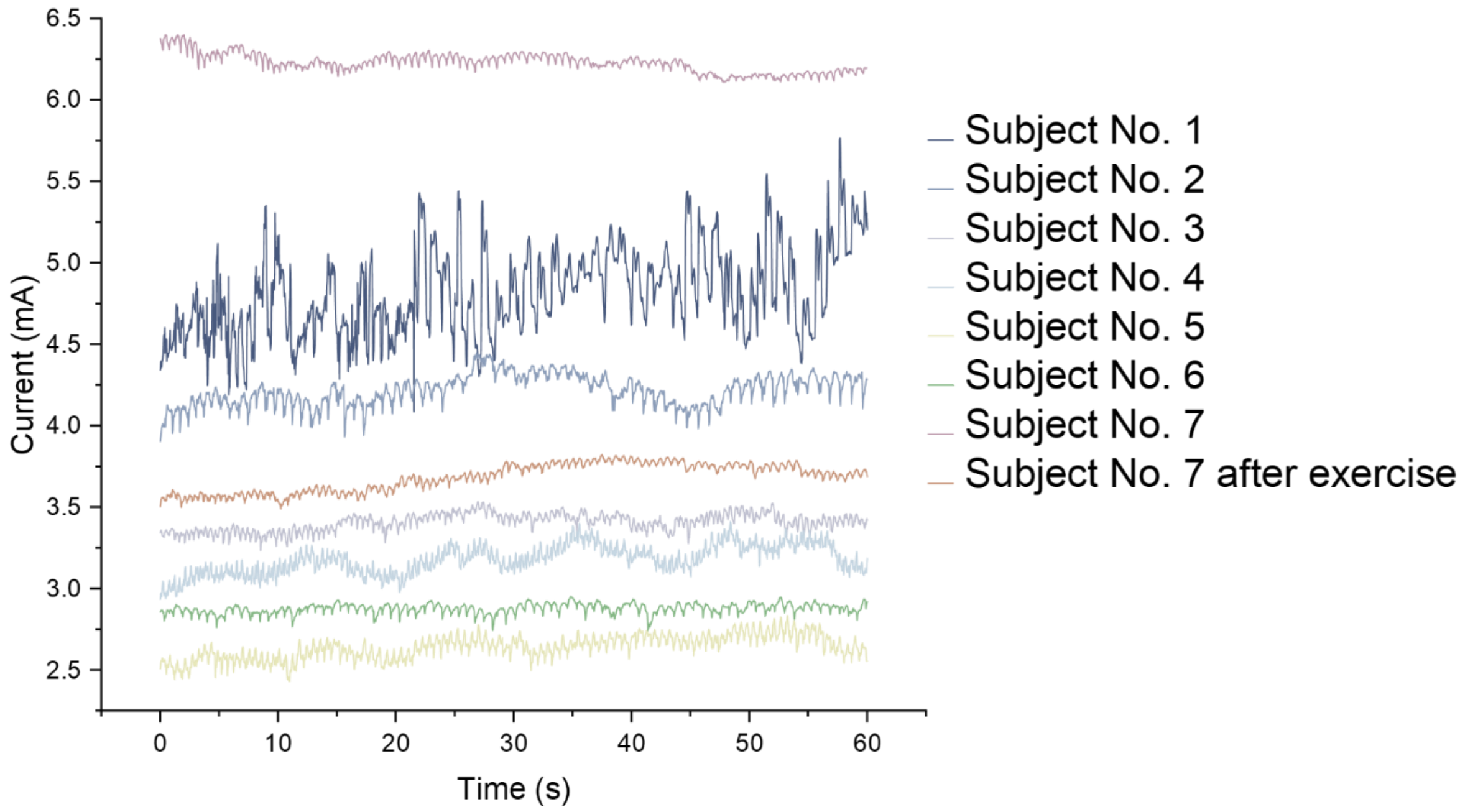

Figure S9. Pulse signals collected from different individuals, showing variations in intensity, frequency, and waveform.

.

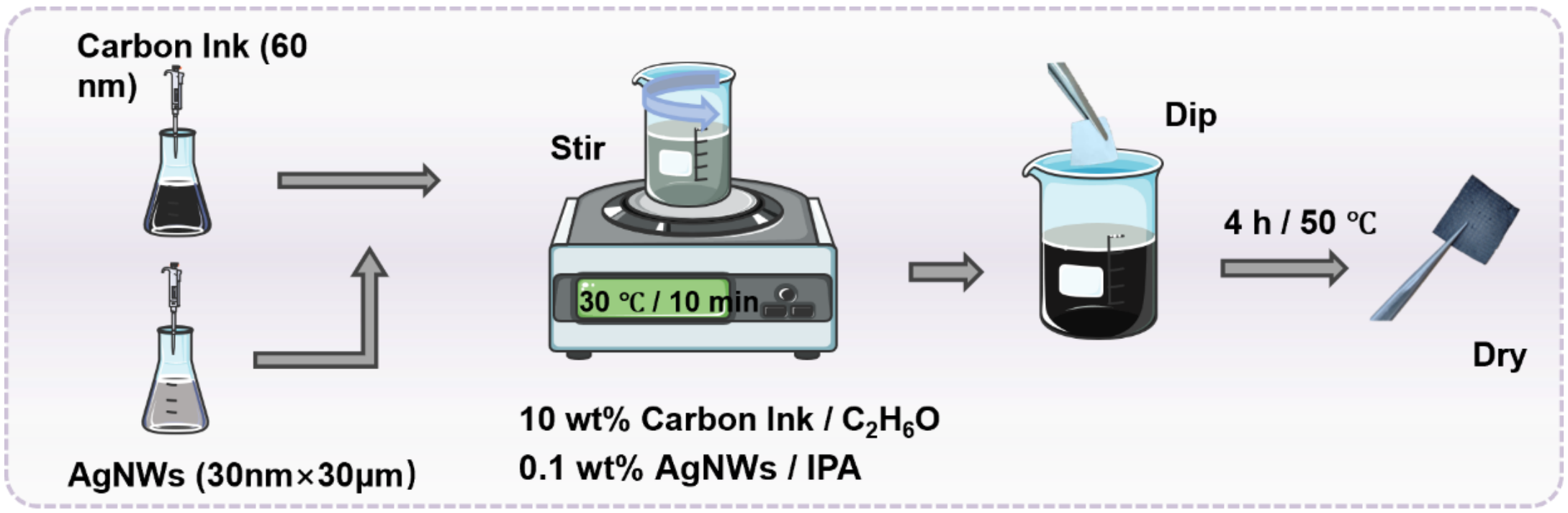

Figure S10. Schematic illustration of the fabrication process of MASW by dip-coating method.

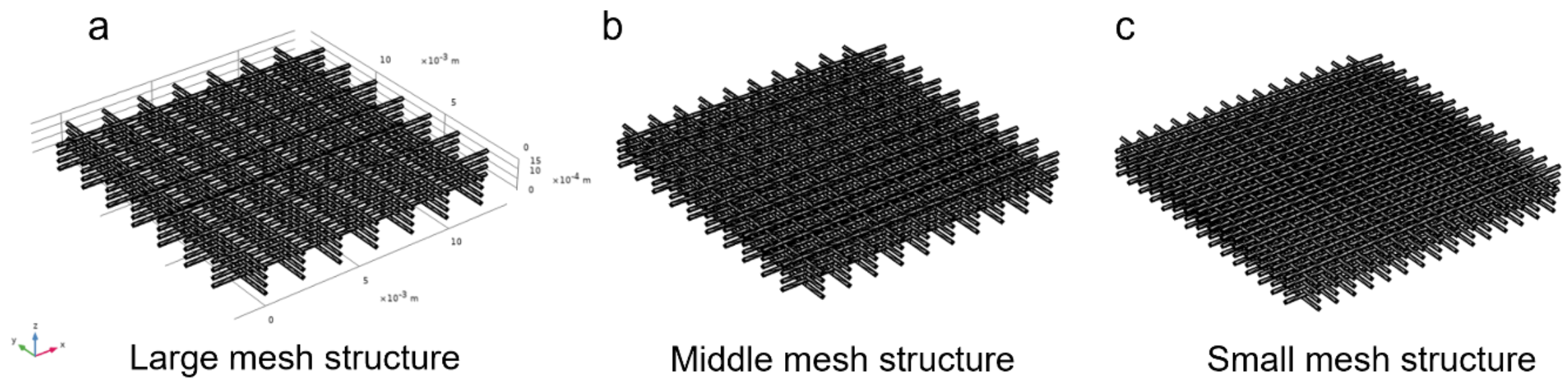

Figure S11. Finite element simulation mesh division of different mesh structures. (a) Large mesh; (b) Middle mesh; (c) Small mesh.

Pressure-sensitive layer	Sensitivity [kPa]$^{-1}$	Linear operating range (kPa)	Reference [1-22]
Mxene/PLA	5.37	0-100	ACS Appl. Mater. Interfaces 15, 5600-5607 (2023)
GGFF	~1	0-10	Nat. Commun. 15, 5040 (2024)
MXene@PU	1.52	0-100	Small Methods 6, e2101051 (2022)
HPM-PDMS	83.9	0-0.14	ACS Appl. Mater. Interfaces 11, 29466-29473 (2019)
CNFs/CNTs	64.23	0-6	ACS Appl. Electron. Mater. 7, 7321-7329 (2025)
PGS	0.76	0-10	Adv. Mater. 27, 6954-6961 (2015)
PDMS/AgNWs	0.02	0-100	Nat. Commun. 16, 2024 (2025)
PDMS/Graphene	18.2	0-80	Adv. Funct. Mater., e10130 (2025)
AgNW	1.5	0-30	ACS Appl. Mater. Interfaces 11, 25034-25042 (2019)
CuNWs/chitosan	1.7	0-5	Org. Electron. 106, 106539 (2022)
POMaC	1.1	0-100	Nat. Electron. 1, 314-321 (2018)
Pectin xerogel	0.03	0-100	Adv. Funct.Mater. 34, 2403268 (2024)
Cellulose/MTS	11.95	0-60	Adv. Funct. Mater. 34, 2410762 (2024)
PVA/AU	0.03	0-100	Science 370, 966-970 (2020)
PDMS:PVA	~10	~0-3	Nat. Commun. 16, 287 (2025)
EGaIn	0.11	0-12.5	Microsyst. Nanoeng. 11, 55 (2025)
P-TENG	9.21	0-8	Nat. Commun. 16, 383 (2025)
prslPDMS	18.94	0-20	Nat. Commun. 14, 1252 (2023)
PSP	4.00	0-100	Adv. Mater. 37, e2410312 (2025)
PCM	0.57	1-13	Wearable Electron. 1, 180-188 (2024)
Paper/Ag	4.3	0-60	npj Flex. Electron. 7, 13 (2023)
LS@CI-AgNPs	4.02	0-50	Small 21, e2407773 (2025)
Interlink FSR 402	0.01 – 0.05	~0.01–100	Interlink Electronics (Commercial)
NextFlex Sensor	~0.02	0–50	NextFlex (Commercial)
StretchSense Sensor	~0.01–0.02	0–100	StretchSense Ltd. (Commercial)
FSRTEK FA402	0.01 – 0.03	0 – 100	FSRTEK (Commercial)
PLA@CI-AgNWs	39.85	0-100	This work

Table S1. Comparison of sensitivity and linearity of reported and commercial flexible pressure sensors.

Sensor number	Mesh structure	Dip-coating Cycles	Conductive fillers	Linearity (R^2)
1	Small (30-mesh)	1	CI	0.8713
2	Small (30-mesh)	2	CI	0.9661
3	Small (30-mesh)	3	CI	0.8871
4	Middle (20-mesh)	1	CI	0.8623
5	Middle (20-mesh)	2	CI	0.9851
6	Middle (20-mesh)	3	CI	0.9552
7	Large (10-mesh)	1	CI	0.9354
8	Large (10-mesh)	2	CI	0.9717
9	Large (10-mesh)	3	CI	0.9926
10	Small (30-mesh)	2	CI	0.9661
11	Small (30-mesh)	2	CI/AgNWs	0.9851
12	Small (30-mesh)	2	AgNWs	0.8070
13	Middle (20-mesh)	2	CI	0.9851
14	Middle (20-mesh)	2	CI/AgNWs	0.9864
15	Middle (20-mesh)	2	AgNWs	0.8466
16	Large (10-mesh)	2	CI	0.9717
17	Large (10-mesh)	2	CI/AgNWs	0.9857
18	Large (10-mesh)	2	AgNWs	0.7693

Table S2. Summary of the linearity of pressure–response curves for MASWs fabricated with different process parameters (mesh structures, dipping cycles, and conductive fillers).

Pressure-sensitive layer	Sensitivity [kPa]$^{-1}$	Response time (ms)	Linear operating range (kPa)	Pressure Cycles	Degradability	Reference [1,15-22]
PSP	4.00	120	0-100	5000	×	Adv. Mater. 37, e2410312 (2025)
PVA/AU	0.03	220	0-100	1000	×	Science 370, 966-970 (2020)
PDMS:PVA	~10	660	~0-3	200	×	Nat. Commun. 16, 287 (2025)
EGaIn	0.11	640	0-12.5	-	×	Microsyst. Nanoeng. 11, 55 (2025)
P-TENG	9.21	70	0-8	2000	×	Nat. Commun. 16, 383 (2025)
prslPDMS	18.94	284	0-20	5000	×	Nat. Commun. 14, 1252 (2023)
PCM	0.57	192	1-13	600	×	Wearable Electron. 1, 180-188 (2024)
Paper/Ag	4.3	330	0-60	4000	√	npj Flex. Electron. 7, 13 (2023)
LS@CI-AgNPs	4.02	<00	0-50	1000	√	Small 21, e2407773 (2025)
PLA@CI-AgNWs	39.85	42	0-100	6000	√	This work

Table S3. Overall performance comparison of flexible pressure sensors, including sensitivity, response time, linear operating range, pressure cycles, and degradability.

Pressure-sensitive layer	Sensitivity $[kPa]^{-1}$	Response time (ms)	Degradability	Reference [2,3,9-22]
PSP	4.00	120	×	Adv. Mater. 37, e2410312 (2025)
PVA/AU	0.03	220	×	Science 370, 966-970 (2020)
PDMS:PVA	~10	660	×	Nat. Commun. 16, 287 (2025)
EGaIn	0.11	640	×	Microsyst. Nanoeng. 11, 55 (2025)
P-TENG	9.21	70	×	Nat. Commun. 16, 383 (2025)
prslPDMS	18.94	284	×	Nat. Commun. 14, 1252 (2023)
PCM	0.57	192	×	Wearable Electron. 1, 180-188 (2024)
PDMS/Graphene	18.20	21	×	Adv. Funct. Mater., e10130 (2025).
PDMS/ AgNWs	0.018	468	×	Nat. Commun. 16, 2024 (2025).
Cellulose/MTS	11.95	93	√	Adv. Funct. Mater. 34, 2410762 (2024).
LS@CI-AgNPs	4.023	<300	√	Small 21, e2407773 (2025)
Paper/Ag	4.30	330	√	npj Flex. Electron. 7, 13 (2023)
POMaC	1.10	1500	√	Nat. Electron. 1, 314-321 (2018)
CuNWs/chitosan	1.70	180	√	Org. Electron. 106, 106539 (2022)
AgNW	1.50	90	√	ACS Appl. Mater. Interfaces 11, 25034-25042 (2019)
Pectin xerogel	0.03	118	√	Adv. Funct.Mater. 34, 2403268 (2024)
PLA@CI-AgNWs	39.85	42	√	This work

Table S4. Comparison of sensitivity and response time of reported conventional and biodegradable flexible pressure sensors

References

1. Wang X*, et al.* Traditional Chinese Medicine (TCM)-Inspired Fully Printed Soft Pressure Sensor Array with Self-Adaptive Pressurization for Highly Reliable Individualized Long-Term Pulse Diagnostics. *Adv. Mater.* **37**, e2410312 (2025).

2. Yang J*, et al.* Interference-free nanogap pressure sensor array with high spatial resolution for wireless human-machine interfaces applications. *Nat. Commun.* **16**, 2024 (2025).

3. He J*, et al.* A High-Performance Pressure Sensor Combining the Biomimetic Structures of Shark and Crocodile Skins. *Adv. Funct. Mater.*, e10130 (2025).

4. Huang J, Xie G, Wei Q, Su Y, Xu X, Jiang Y. Degradable MXene-Doped Polylactic Acid Textiles for Wearable Biomonitoring. *ACS Appl. Mater. Interfaces* **15**, 5600-5607 (2023).

5. Wang K*, et al.* Multispecies-coadsorption-induced rapid preparation of graphene glass fiber fabric and applications in flexible pressure sensor. *Nat. Commun.* **15**, 5040 (2024).

6. Wei Q*, et al.* MXene-Sponge Based High-Performance Piezoresistive Sensor for Wearable Biomonitoring and Real-Time Tactile Sensing. *Small Methods* **6**, e2101051 (2022).

7. Zhao T, Li T, Chen L, Yuan L, Li X, Zhang J. Highly Sensitive Flexible Piezoresistive Pressure Sensor Developed Using Biomimetically Textured Porous Materials. *ACS Appl. Mater. Interfaces* **11**, 29466-29473 (2019).

8. Dai J, Li H, Xie G, Que L, Yuan H, Su Y. Self-Supported Composite Textiles for Ultrasensitive Physiological Monitoring. *ACS Appl. Electron. Mater.* **7**, 7321-7329 (2025).

9. Boutry CM, Nguyen A, Lawal QO, Chortos A, Rondeau-Gagne S, Bao Z. A Sensitive and Biodegradable Pressure Sensor Array for Cardiovascular Monitoring. *Adv. Mater.* **27**, 6954-6961 (2015).

10. Gao L*, et al.* All Paper-Based Flexible and Wearable Piezoresistive Pressure Sensor. *ACS Appl. Mater. Interfaces* **11**, 25034-25042 (2019).

11. Song Z*, et al.* Biodegradable and flexible capacitive pressure sensor for electronic skins. *Org. Electron.* **106**, 106539 (2022).

12. Boutry CM*, et al.* A stretchable and biodegradable strain and pressure sensor for orthopaedic application. *Nat. Electron.* **1**, 314-321 (2018).

13. Basarir F*, et al.* Edible and Biodegradable Wearable Capacitive Pressure Sensors: A Paradigm Shift toward Sustainable Electronics with Bio-Based Materials. *Adv. Funct. Mater.* **34**, 2403268 (2024).

14. Li A*, et al.* All-Paper-Based, Flexible, and Bio-Degradable Pressure Sensor with High Moisture Tolerance and Breathability Through Conformally Surface Coating. *Adv. Funct. Mater.* **34**, 2410762 (2024).

15. Lee S*, et al.* Nanomesh pressure sensor for monitoring finger manipulation without sensory interference. *Science* **370**, 966-970 (2020).

16. Shang J*, et al.* A flexible catheter-based sensor array for upper airway soft tissues pressure monitoring. *Nat. Commun.* **16**, 287 (2025).

17. Wang Q*, et al.* Multimodal response characteristics of convective liquid metal sensitive layers in flexible pressure sensor. *Microsyst. Nanoeng.* **11**, 55 (2025).

18. Liu Y*, et al.* Triboelectric tactile sensor for pressure and temperature sensing in high-temperature applications. *Nat. Commun.* **16**, 383 (2025).

19. Zhang Y*, et al.* Localizing strain via micro-cage structure for stretchable pressure sensor arrays with ultralow spatial crosstalk. *Nat. Commun.* **14**, 1252 (2023).

20. Huang J*, et al.* Hierarchical carbon nanotube-decorated polyacrylonitrile smart textiles for wearable biomonitoring. *Wearable Electron.* **1**, 180-188 (2024).

21. Wei Y*, et al.* Fully paper-integrated hydrophobic and air permeable piezoresistive sensors for high-humidity and underwater wearable motion monitoring. *npj Flex. Electron.* **7**, 13 (2023).

22. Dai J, Xie G, Huo X, Li J, Deng S, Su Y. Implantable and Biodegradable Smart Textiles for Continuous Limb and Gastrointestinal Motility Monitoring. *Small* **21**, e2407773 (2025).
