# Supplementary material for: Multiscale artificial spider web for comprehensive pressure sensing and human-machine interaction
Source: Nat Commun. 2026 Jul 4;17:8316. doi: 10.1038/s41467-026-74200-y (PMC13470477; doi:10.1038/s41467-026-74200-y)
Supplement: Supplementary file 2 [file 41467_2026_74200_MOESM2_ESM.pdf]

Description of Additional Supplementary Files

File name: Movie S1
Description: Breathability test of MASWs with three different mesh structures.

File name: Movie S2
Description: Finite element simulation of stress distribution of three mesh structures.

File name: Movie S3
Description: Single-finger control of robotic hand with adjustable grip force.

File name: Movie S4
Description: Complex gesture control of robotic hand with five sensors.
