# Supplementary material for: Multiscale artificial spider web for comprehensive pressure sensing and human-machine interaction
Source: Nat Commun. 2026 Jul 4;17:8316. doi: 10.1038/s41467-026-74200-y (PMC13470477; doi:10.1038/s41467-026-74200-y)
Supplement: Supplementary file 3 [file 41467_2026_74200_MOESM7_ESM.pdf]

nature portfolio

Peer Review File

Multiscale Artificial Spider Web for Comprehensive Pressure Sensing and Human-Machine Interaction

Corresponding Author: Professor Tae-Woo Lee

This file contains all reviewer reports in order by version, followed by all author rebuttals in order by version.

Version 0:

Reviewer comments:

Reviewer #1

(Remarks to the Author)
Authors proposed a multiscale conductive three-dimensional network inspired by natural spider webs for pressure sensing. The sensos has high sensitivity, rapid response, reliable mechanical integrity, and long linear sensing range from 0-100 kPa. The sensor is simple but useful. They also developed a human–machine interaction system to supervise Parkinson's disease. The manuscript has merit. I suggest major revision.
1. The novelty of the manuscript should be further emphasized.
2. The mechanism of the long linear sensing range, high sensitivity and rapid response should be explained more clearly.
3. Authors describe the sensor has reliable mechanical integrity, namely mechanical resistance. In that case, the morphological characteristics of sensor (SEM images) before and after testing of 3000 or longer loading cycles under the pressures (in the range of 0-100 kPa) should be provided.
4. The description of the deep learning framework (Transformer) in the manuscript should be more specific, to help readers' understanding of the model's design and reproducibility of the results. How many samples were used in total? Sample size of the training set and testing set should be provided. Too small dataset risks overfitting. Key training parameters (e.g., learning rate, batch size, optimization algorithm, number of training epochs, loss function type) should also be explained.

Reviewer #2

(Remarks to the Author)
Report on: Multiscale Artificial Spider Web for Comprehensive Pressure Sensing and Human-Machine Interaction.
by jing Dai et al.

The authors focused on the development of a pressure sensing solution inspired by natural spider webs to overcome some open challenges in this scenario. Then, they proposed a case study in the field of rehabilitation for Parkinson's disease. The application (both pressure sensing and fine motor control in the rehabilitation process) has an immense potential, and it is very challenging to design these sensors that fulfill the strict requirements to be applied in the proposed scenario of interest from a technological and metrological point of view.
Overall, the manuscript is interesting and well structured; however, I have several concerns that should be addressed:

1. Lines 65-69. "As a result, the MASW exhibits exceptional overall performance among pressure-resistive sensors, simultaneously achieving ultrahigh sensitivity (39.85 kPa-1), rapid response time (42 ms), excellent cyclic stability over 6000 loading cycles, and long-term durability exceeding 100 days, which far exceeds commercial sensors that mostly use the operating range of 0-100 kPa.". It is not clear the relation between the first part of the sentence and the second part (which far exceeds commercial sensors that mostly use the operating range of 0-100 kPa). Please clarify.

2. Line 176 (and in some other parts of the manuscript). The sensitivity is not constant; please explain that it is a mean sensitivity considered equal to the slope of the best-fitting line.

3. Line 182 (and in some other parts of the manuscript). Please highlight that 42 ms is the rise time (it can be confused with the time constant of the system).

4. Line 205 (and in some other parts of the manuscript). Why did you consider a pressure range of 0-100 kPa? Is it motivated by the application of interest?.

5. In the tables S2 and S3, you reported the response time that is different from the rise time. Please use the same parameter to compare the dynamic response of the proposed sensor with the sensors reported in the literature. This is crucial to compare your results with literature.

6. Line 282. The application of interest needs an accurate measure of pressure. Why did you use a voltage divider to convert R into V? When accurate measurements are required, it is much better to use a Wheatstone bridge.

Version 1:

Reviewer comments:

Reviewer #1

(Remarks to the Author)
Accept

Reviewer #2

(Remarks to the Author)
The paper can be accepted in the current form

Open Access This Peer Review File is licensed under a Creative Commons Attribution 4.0 International License, which permits use, sharing, adaptation, distribution and reproduction in any medium or format, as long as you give appropriate credit to the original author(s) and the source, provide a link to the Creative Commons license, and indicate if changes were made.
In cases where reviewers are anonymous, credit should be given to 'Anonymous Referee' and the source.
The images or other third party material in this Peer Review File are included in the article's Creative Commons license, unless indicated otherwise in a credit line to the material. If material is not included in the article's Creative Commons license and your intended use is not permitted by statutory regulation or exceeds the permitted use, you will need to obtain permission directly from the copyright holder.
To view a copy of this license, visit https://creativecommons.org/licenses/by/4.0/

Responses to Reviewer #1:

Authors proposed a multiscale conductive three-dimensional network inspired by natural spider webs for pressure sensing. The sensos has high sensitivity, rapid response, reliable mechanical integrity, and long linear sensing range from 0-100 kPa. The sensor is simple but useful. They also developed a human–machine interaction system to supervise Parkinson's disease. The manuscript has merit. I suggest major revision.

Response)

We sincerely thank the reviewer for the valuable and constructive comments on our work. In response, we have further strengthened the novelty of this study in the revised manuscript in three key aspects.

First, the sensing mechanism of the MASW has been clarified in greater depth by integrating Hertzian contact theory, percolation theory, and tunneling conduction, which together explain the origin of the long linear sensing range, high sensitivity, and rapid response of the device.

Second, we have supplemented new experimental evidence to further support the reliability of the sensor. Specifically, SEM characterization of the MASW before and after 6000 pressure loading cycles has been added to demonstrate its excellent mechanical durability from a microscopic perspective.

Third, we further improved the readability and reproducibility of the manuscript by providing a more detailed description of the parameters used in the neural network model.

We believe that these revisions enhance the scientific rigor and clarity of the manuscript.

Comment 1. *The novelty of the manuscript should be further emphasized.*

Response)

We thank the reviewer for this helpful suggestion. In the revised manuscript, we have further clarified and emphasized the key novelties and contributions of this work.

The main innovations of this study can be summarized as follows:

1. **Bioinspired structural design and mechanism analysis.**

Unlike conventional planar or randomly structured (single-scale) pressure sensors that enhance mostly single performance parameter, the proposed structure introduces a spider-web-inspired *multiscale* conductive three-dimensional (3D) network that simultaneously enables efficient stress transmission and enhanced mechanical stability. In addition, the sensing mechanism is systematically elucidated by integrating Hertzian contact theory, percolation theory, and tunneling conduction, providing a clear physical understanding of the pressure-dependent electrical response.

2. **Outstanding sensing performance with balanced mechanical robustness.**

Conventional flexible pressure sensors typically suffer from a trade-off between sensitivity and mechanical stability. In contrast, the proposed MASW achieves excellent overall performance, including high sensitivity (39.85 $[kPa]^{-1}$), fast response (42 ms), outstanding durability over 6000 loading cycles, and long-term operational stability exceeding 100 days, demonstrating a combination of performance metrics that is rarely achieved simultaneously in previously reported systems.

3. **System-level demonstration of practical wearable sensing and human–machine interaction.**

Beyond device-level performance, this work further demonstrates system-level integration, including neural-network assisted recognition of physiological signals (pulse, respiration, and phonation) and robotic hand control for Parkinson's disease, highlighting the practical applicability of the proposed architecture in intelligent healthcare and human–machine interaction.

To highlight these contributions more clearly, we have revised the Introduction and Discussion sections to explicitly summarize the novelty and significance of this work.

Revised parts in the manuscript)

- manuscript on page 3

"In this work, inspired by the architecture of natural spider webs, we propose and construct a flexible pressure sensor that integrates a multiscale conductive three-dimensional (3D) network. The multiscale artificial spider web (MASW) is fabricated via a copper-mesh-assisted electrospinning process of polylactic acid (PLA), where the resulting electrospun fiber network markedly enhances stress transmission while the embedded mesh skeleton reinforces mechanical stability. Furthermore, coating with conductive nanoparticles and nanowires establishes a core–shell–network dual microstructure on the PLA matrix, enabling 3D conductive contact in the sensing layer to significantly enhance sensitivity and response speed. The sensing mechanism is further analyzed based on contact mechanics, percolation behavior, and tunneling conduction, providing a clear physical understanding of the pressure-dependent electrical response. As a result, the MASW exhibits exceptional overall performance among pressure-resistive sensors, simultaneously achieving ultrahigh sensitivity (39.85 $[kPa]^{-1}$) within 0–100 kPa (The sensitivity far exceeding the commercial sensors (~0.05 $[kPa]^{-1}$) for the same pressure range), rapid response time (42 ms), excellent cyclic stability over 6000 loading cycles, and long-term durability exceeding 100 days. Intrinsic biodegradability allows safe disposal, mitigating environmental risks of conventional flexible devices. With conformal attachment to the body, the sensor enables real-time monitoring of multiple physiological signals (pulse,

breathing and vocalization) and, when combined with neural-network recognition, achieves precise classification of multiple pulse and laryngeal movements. Moreover, by integrating MASWs into robotic hand, a human–machine interaction system was established to potentially restore fine motor control in Parkinson's disease patients, highlighting the sensor's applicability in intelligent healthcare and IoT interfaces."

- manuscript on page 16

"This study presented an artificial spider web (MASW) that overcomes the performance trade-off in conventional pressure sensors through a multiscale core-shell-network architecture with a 3D fiber skeleton inspired by natural spider webs. The MASW transduces external pressure into electrical signals via its three-dimensional interconnected network, where efficient stress transmission and multidimensional conductive pathways ensure both high sensitivity and rapid response. Simultaneously achieving ultrahigh sensitivity (39.85 $[kPa]^{-1}$), fast response (42 ms), and remarkable durability (6000 cycles, 100 days), the MASW fully cover the detection ranges of all major physiological signals. This capability allows real-time monitoring of diverse physiological signals, including pulse, breathing, vocalization, and joint movements and such a combination of performance metrics is rarely achieved in previously reported flexible pressure sensors.

Assisted by a transformer-based neural network, it achieves precise recognition and classification of these physiological signals while maintaining eco-friendly biodegradability. Furthermore, the MASW-based adaptive robotic hand control system (from adjustable grasping to complex gesture recognition) demonstrates promising potential for assisting fine motor rehabilitation in patients with Parkinson's disease. In addition, a wireless sensor network enables remote signal transmission and real-time visualization, overcoming spatial constraints of traditional wired systems. Collectively, this work highlights MASW as a promising route toward sustainable flexible electronics for smart healthcare, personalized prosthetics, and next-generation human–machine interfaces."

Comment 2. *The mechanism of the long linear sensing range, high sensitivity and rapid response should be explained more clearly.*

Response)

We thank the reviewer for this valuable suggestion. In the revised manuscript, we have substantially expanded and clarified the sensing mechanism, with particular emphasis on the physical origin of the long linear sensing range, high sensitivity, and rapid response of the MASW.

Specifically, the resistance variation of the device is dominated by pressure-induced changes in the contact resistance between conductive units, which follow Hertzian contact mechanics[R1]. The applied pressure enlarges the effective contact area between conductive fillers, leading to a reduction in contact resistance.

Furthermore, the core-shell-network structure forms multiscale conductive pathways, including point contacts, line contacts, and surface contacts. These hierarchical contacts significantly increase the number of conductive junctions and enhance the modulation of conductive pathways under external pressure.

The electrical behavior of the system also follows percolation theory [R2]. Compression of the fiber network increases the local filler packing density, resulting in a continuous evolution of the conductive network and thus contributing to the *high sensitivity of the sensor*.

In addition, nanoscale gaps between conductive particles allow electron transport via tunneling conduction, which enables exponential resistance modulation under small pressure stimuli and further enhances *sensitivity in the low-pressure region* [R3].

Importantly, the multiscale micro/nanostructures allow progressive contact evolution from point to line to surface contacts under compression, preventing early conductive saturation and enabling *a broad and nearly linear sensing range*. Meanwhile, the elastic fiber framework facilitates rapid deformation and recovery, leading to fast formation and separation of conductive contacts and thereby contributing to the *rapid response* of the device. Figure 1b also illustrates the sensing mechanism of MASW.

These explanations have been incorporated into the revised manuscript to clarify the sensing mechanism.

Revised parts in the manuscript)

- manuscript on page 6

"The overall resistance of MASW can be expressed as

$$R = R_{bulk} + R_{contact}$$

where R_{bulk} represents the intrinsic resistance of the conductive fillers and $R_{contact}$ denotes the interfacial contact resistance between conductive units. In this system, the pressure-dependent resistance variation is primarily dominated by changes in contact resistance.

According to Hertzian contact theory, when two elastic bodies are subjected to a normal force F, the contact radius a can be described as

$$a = \left(\frac{3FR}{4E^*}\right)^{1/3}$$

where R is the equivalent curvature radius and E^* is the effective elastic modulus. Since the contact resistance is inversely proportional to the contact area, $R_{contact} \propto \frac{1}{a^2}$, the contact resistance decreases with increasing pressure following

$$R_{contact} \propto F^{-2/3}$$

Consequently, the applied pressure enlarges the effective contact area between conductive units, resulting in a reduction in overall device resistance.

Compared with conventional single-filler systems, the core–shell–network structure in MASW establishes multiscale conductive pathways, including surface contacts (CI shell layers),

line contacts (AgNW bridges), and point contacts (carbon particles). The coexistence of these contact modes significantly increases the number of conductive junctions per unit volume and amplifies pressure-induced variations in conductive pathways.

The electrical behavior further follows percolation theory. When the conductive filler fraction φ exceeds the critical percolation threshold φ_c, the conductivity follows

$$\sigma \propto (\varphi - \varphi_c)^t$$

During compression, deformation of the framework increases the local filler packing density ($\varphi = \varphi_0 + \Delta\varphi(P)$), resulting in a progressive increase in conductivity. The CI/AgNWs hybrid filler effectively lowers the percolation threshold, allowing the conductive network to evolve continuously under pressure.

In addition, nanoscale gaps between conductive particles enable electron transport through tunneling conduction, with the tunneling resistance expressed as

$$R_t \propto e^{\beta d}$$

where d represents the interparticle distance. External pressure reduces the distance, leading to an exponential decrease in resistance and enabling high sensitivity under small pressure stimuli.

Importantly, the three-dimensional multiscale structure enables progressive contact evolution during compression, where conductive junctions gradually transition from point contacts to line contacts and eventually to surface contacts. This gradual formation of conductive pathways prevents premature conductive saturation and ensures a stable and nearly linear resistance variation over a broad pressure range. Meanwhile, the elastic fiber framework facilitates rapid mechanical deformation and recovery, allowing fast formation and separation of conductive contacts, which enabled highly sensitive detection of subtle pressure and rapid dynamic response on the millisecond timescale."

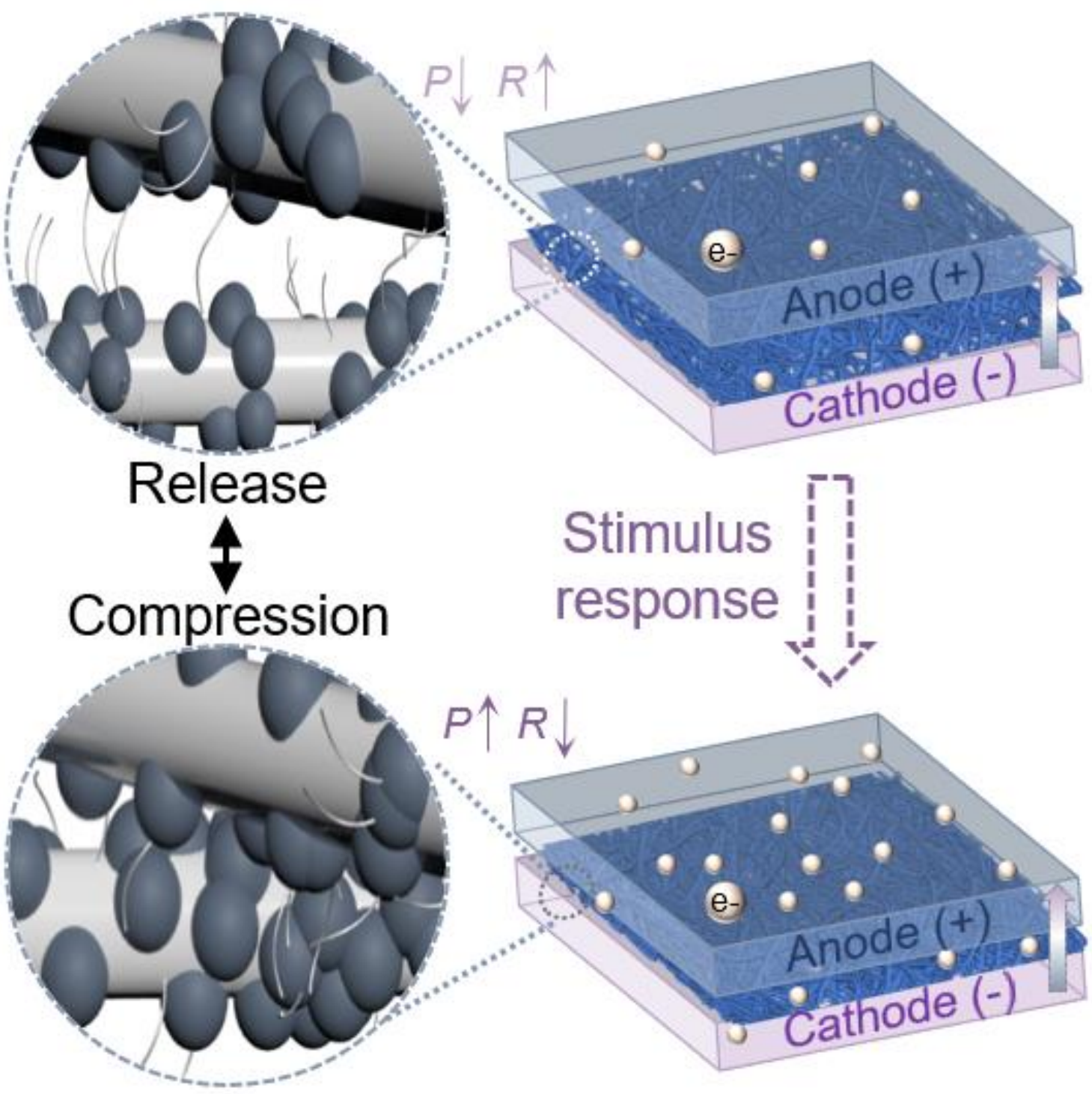

Figure 1. (b) Schematic diagram of MASW sensing mechanism

Comment 3. *Authors describe the sensor has reliable mechanical integrity, namely mechanical resistance. In that case, the morphological characteristics of sensor (SEM images) before and after testing of 3000 or longer loading cycles under the pressures (in the range of 0-100 kPa) should be provided.*

Response)

We sincerely thank the reviewer for the valuable suggestion. We have supplemented the manuscript with additional SEM characterizations of the MASW (with a small mesh skeleton subjected to two dip-coating cycles of CI/AgNWs, which are relevant to physiological monitoring and human–machine interaction applications.) before and after 6000 pressure loading cycles,

The SEM cross-sectional images (Figure S7) show no significant morphological differences after cyclic loading. The only observable change is a slight thickness reduction of 4.88 μm after 6000 cycles. This minimal deformation further confirms the reliable mechanical integrity and excellent durability of the MASW. The manuscript has been revised accordingly.

Revised parts in the manuscript)

- manuscript on page 12

MASW also demonstrated robust durability, with no significant changes observed in its microscopic morphology after 6000 loading cycles, exhibiting only a minimal thickness variation of 4.88 μm (Figure S7). Meanwhile, the resistance variation remained below 2.26%, (Figure S8, 4g, 4h) further confirming its outstanding mechanical stability and electrical reliability.

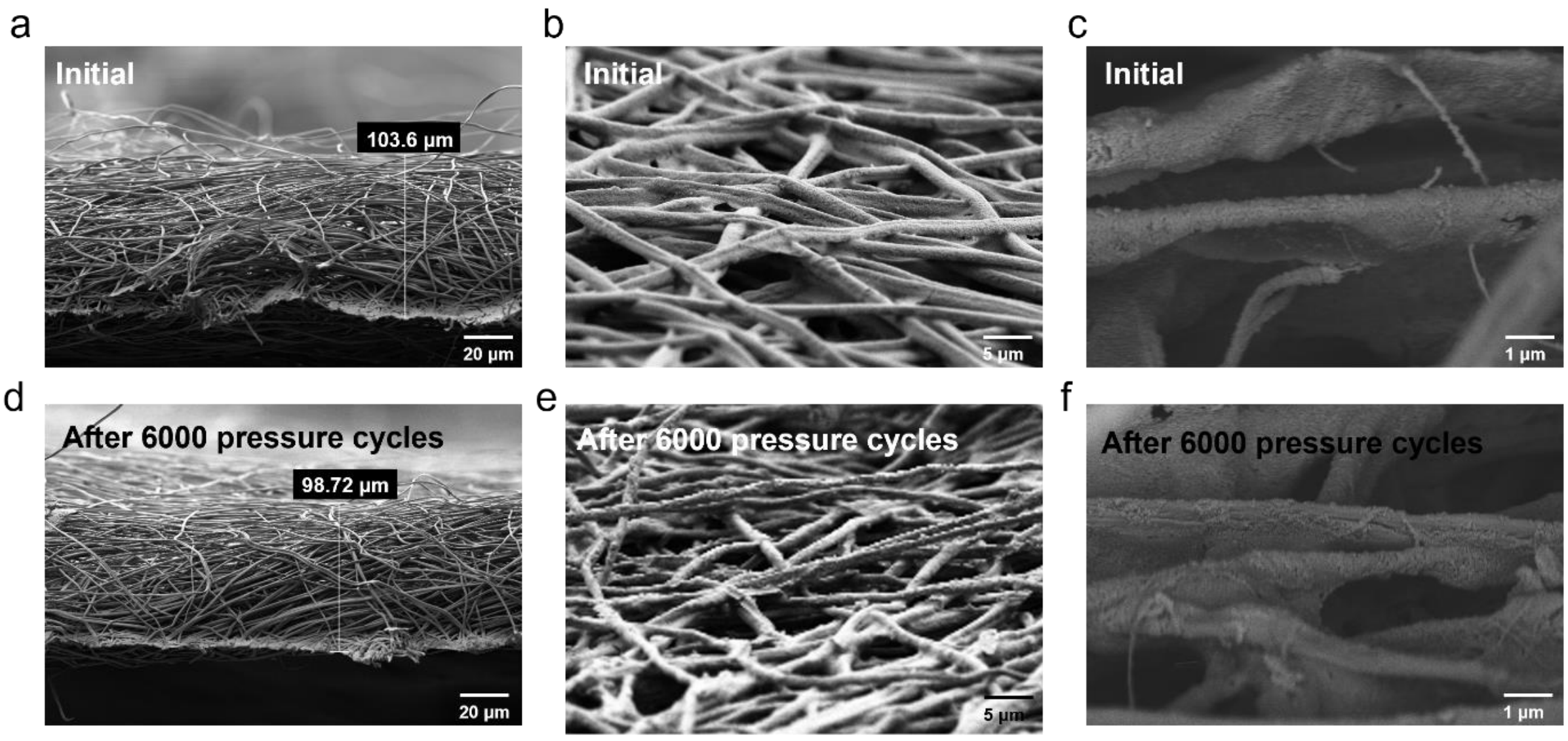

Figure S7. (a-c) SEM cross-sectional characterization of MASW at different scales in the initial state; (b) SEM cross-sectional characterization of MASW at different scales after 6000 cycles of loading pressure.

Comment 4. *The description of the deep learning framework (Transformer) in the manuscript should be more specific, to help readers' understanding of the model's design and reproducibility of the results. How many samples were used in total? Sample size of the training set and testing set should be provided. Too small dataset risks overfitting. Key training parameters (e.g., learning rate, batch size, optimization algorithm, number of training epochs, loss function type) should also be explained.*

Response)

We thank the reviewer for this insightful comment.

The Transformer model captures long-range dependencies in time-series pressure signals via self-attention, enabling accurate extraction of subtle temporal patterns that are difficult to resolve using conventional convolutional or recurrent approaches.

To improve clarity and reproducibility, we have supplemented detailed descriptions of the dataset size and key training parameters in the Methods section and hope this addresses the concerns of the reviewer [R4]. Figure 5b also presents the basic architecture of the neural network model. The revised manuscript is shown below.

Revised parts in the manuscript)

- manuscript on page 13

"Leveraging the excellent sensing capabilities of MASW, we further developed a neural network using the Transformer model for intelligent processing of the acquired signals and precise evaluation of human physiological states (Figure 5b)[46]. The neural network is trained by inputting large volumes of pressure signal data, after which it can accurately recognize and classify different pressure signal. The Transformer model captures long-range dependencies in time-series pressure signals via self-attention, enabling accurate extraction of subtle temporal patterns that are difficult to resolve using conventional convolutional or recurrent approaches."

- manuscript on page 19

"Network Model

A neural network model using the Transformer architecture was employed. The Transformer relies entirely on self-attention mechanisms to model input and output waveform representations, without using sequence-aligned recurrent neural networks (RNNs) or convolutional operations. This design enables efficient modeling of long-range dependencies while allowing parallel computation during training, thereby improving computational efficiency.

Raw signals were first interpolated to ensure a uniform number of data points across different samples. Subsequently, dimensionality reduction was performed using an autoencoder to extract compact latent representations and reduce redundancy in the original feature space. The compressed features were then fed into a softmax classifier for pressure signal recognition and movement state identification.

For pulse signal monitoring and recognition, the classifier consisted of one input layer (200 neurons), two hidden layers (50 and 20 neurons), and one output layer (8 neurons). Eight distinct pulse categories were included, with 300 samples collected per category, resulting in a total of 2400 samples.

For laryngeal movement monitoring and recognition, the classifier included one input layer (100 neurons), two hidden layers (50 and 20 neurons), and one output layer (6 neurons). Six different laryngeal movement categories were involved, with 300 samples per category, yielding a total of 1800 samples.

The activation function used in all hidden layers was the Rectified Linear Unit (ReLU), while the output layer employed the softmax activation function to generate probability distributions over the target classes.

The dataset was randomly divided using stratified sampling, with 80% of the data used for training (cross-validation) and 20% reserved for independent testing. For pulse recognition, this corresponds to 1920 training samples and 480 testing samples. For laryngeal movement recognition, 1440 samples were used for training and 360 for testing.

The model was trained using the Adam optimizer with an initial learning rate of 1×10^{-3}. The batch size was set to 32, and the maximum number of training epochs was 300. Categorical cross-entropy was adopted as the loss function. Early stopping (patience = 30 epochs) was applied to prevent overfitting, and the learning rate was adaptively reduced using a ReduceLROnPlateau strategy (reduction factor = 0.5, minimum learning rate = 1×10^{-6}).

To further mitigate overfitting due to the relatively moderate dataset size, dropout regularization (rate = 0.3-0.4) and batch normalization were incorporated into the network.

All experiments were conducted with a fixed random seed to ensure reproducibility.”

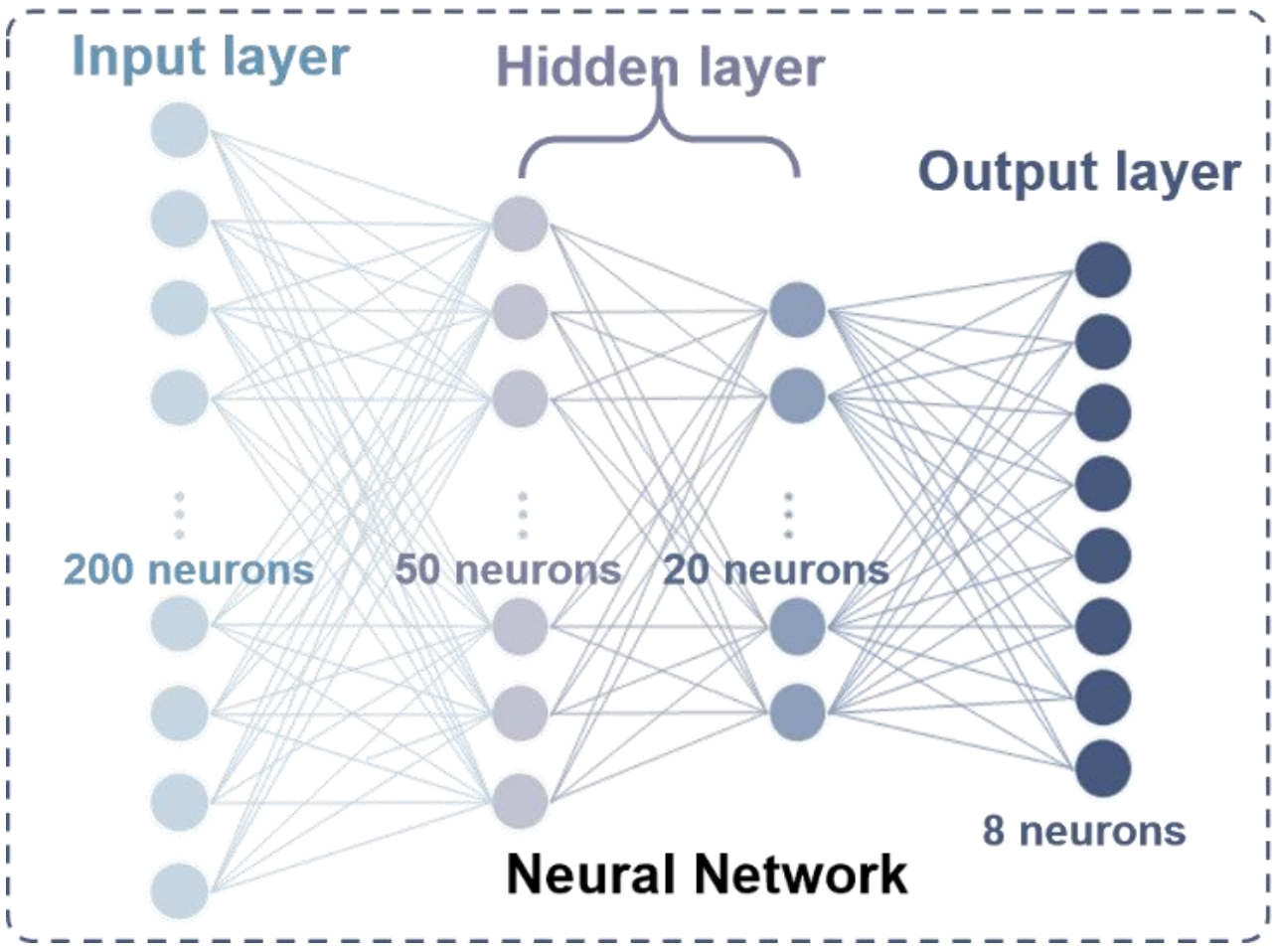

Figure 5. (b) The components of neural network

Responses to Reviewer #2:

The authors focused on the development of a pressure sensing solution inspired by natural spider webs to overcome some open challenges in this scenario. Then, they proposed a case study in the field of rehabilitation for Parkinson's disease. The application (both pressure sensing and fine motor control in the rehabilitation process) has an immense potential, and it is very challenging to design these sensors that fulfill the strict requirements to be applied in the proposed scenario of interest from a technological and metrological point of view.

Overall, the manuscript is interesting and well structured; however, I have several concerns that should be addressed:

Response)

We sincerely thank the reviewer for the valuable comments and suggestions regarding our work. Based on these comments, several revisions have been made to improve the clarity and readability of the manuscript as well as technical rigor.

Specifically, ambiguous or potentially misleading expressions in the original manuscript have been carefully revised. In addition, a new section entitled "Parameter Definition" has been added to systematically define key parameters such as sensitivity and response time. We have also carefully examined and corrected the issues pointed out by the reviewer throughout the manuscript. Furthermore, we have clarified the rationale for selecting the 0-100 kPa pressure detection range and provided a detailed comparison between the voltage divider and the Wheatstone bridge configurations in the human–machine interaction system developed in this work.

We hope that these revisions and explanations adequately address the reviewer's concerns and further improve the clarity and overall quality of the manuscript.

Comment 1. *Lines 65-69. "As a result, the MASW exhibits exceptional overall performance among pressure-resistive sensors, simultaneously achieving ultrahigh sensitivity (39.85 kPa-1), rapid response time (42 ms), excellent cyclic stability over 6000 loading cycles, and long-term durability exceeding 100 days, which far exceeds commercial sensors that mostly use the operating range of 0-100 kPa.". It is not clear the relation between the first part of the sentence and the second part (which far exceeds commercial sensors that mostly use the operating range of 0-100 kPa). Please clarify.*

Response)

We appreciate the reviewer's observation. We recognize that the original statement could potentially cause misunderstanding. Our intention was to indicate that the MASW exhibits significantly higher sensitivity than commercial sensors operating within the same 0–100 kPa detection range (as shown in Figure 4c and Table S1).

To improve precision, we have revised the statement as follows:

Revised parts in the manuscript)

- manuscript on page 4

"As a result, the MASW exhibits exceptional overall performance among pressure-resistive sensors, simultaneously achieving ultrahigh sensitivity (39.85 $[kPa]^{-1}$) within 0-100 kPa (The sensitivity far exceeding the commercial sensors (~0.05 $[kPa]^{-1}$) for the same pressure range), rapid response time (42 ms), excellent cyclic stability over 6000 loading cycles, and long-term durability exceeding 100 days."

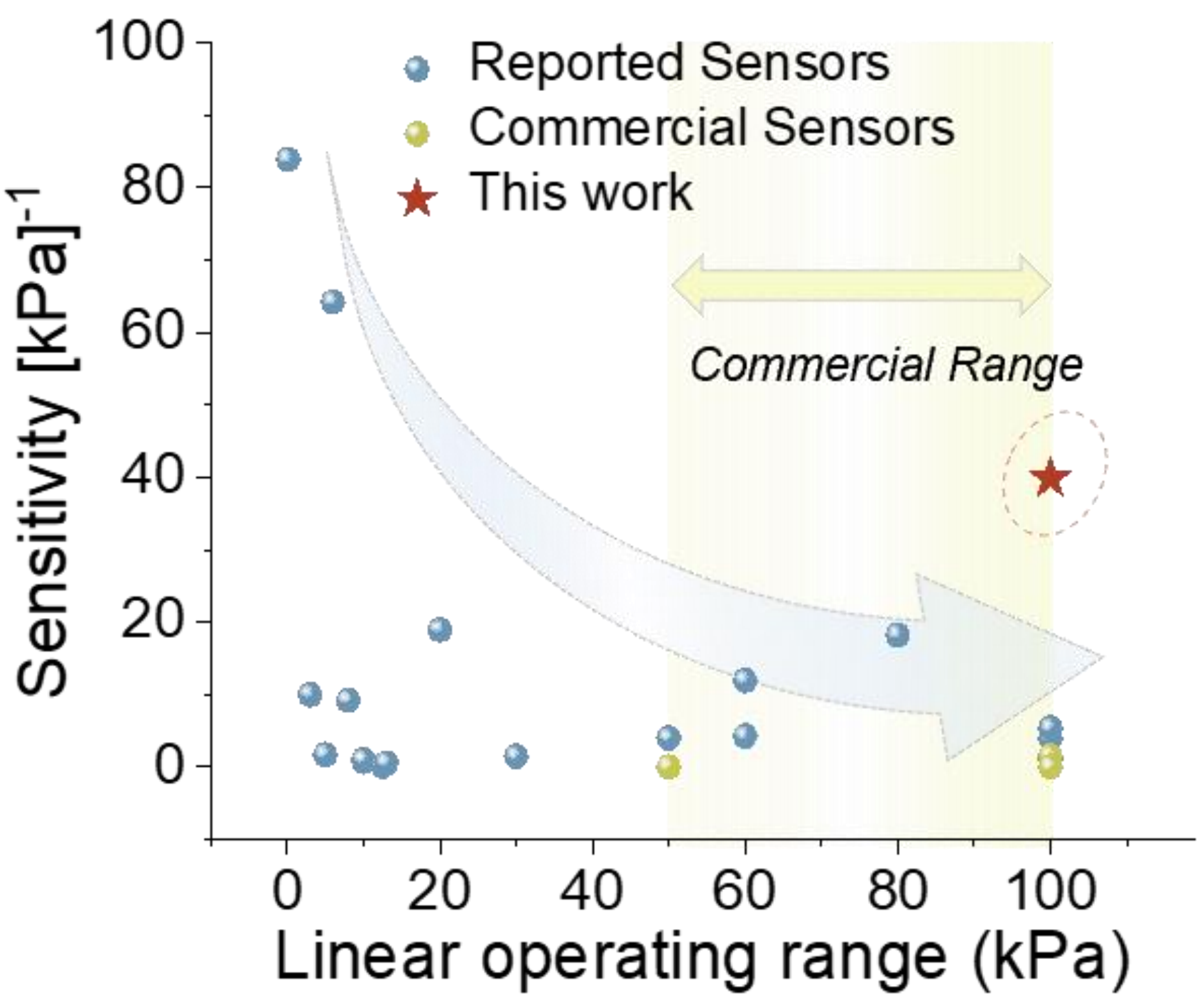

Figure 4. (c) Comparison of sensitivity and linear operating range with reported and commercial flexible pressure sensors

Pressure-sensitive layer	Sensitivity [kPa]$^{-1}$	Linear operating range (kPa)	Reference
…	…	…	…
Interlink FSR 402	0.01 – 0.05	~0.01–100	Interlink Electronics (Commercial)
NextFlex Sensor	~0.02	0–50	NextFlex (Commercial)
StretchSense Sensor	~0.01–0.02	0–100	StretchSense Ltd. (Commercial)
FSRTEK FA402	0.01 – 0.03	0 – 100	FSRTEK (Commercial)
PLA@CI-AgNWs	39.85	0-100	This work

Table S1. Comparison of sensitivity and linearity of reported and commercial flexible pressure sensors.

Comment 2. *Line 176 (and in some other parts of the manuscript). The sensitivity is not constant; please explain that it is a mean sensitivity considered equal to the slope of the best-fitting line.*

Response)

The sensitivity reported in this work refers to the normalized sensitivity, defined as:

$$S = \frac{\Delta I/I_0}{\Delta P}$$

Where ΔI is the relative current variation, I_0 is the initial current without applied pressure, and ΔP is the applied pressure variation.

Indeed, the sensitivity is not a constant value over the entire pressure range due to the nonlinear contact evolution and conductive pathway reconstruction under compression.

In this work, the reported sensitivity represents the average sensitivity within the 0-100 kPa pressure range, obtained from the slope of the best linear fitting of the corresponding pressure–response curve, with the linearity quantified by the coefficient of determination (R^2). For example, as shown in Figure 4b, the MASW exhibits a sensitivity of 39.85 $[kPa]^{-1}$ corresponding to the slope of the fitted line of its pressure–response curve within 0-100 kPa, with a linearity of R^2=0.9851.

This definition enables a consistent and quantitative comparison with previously reported sensors [R5, R6, R7, R8]. To improve clarity and reproducibility, we have added a dedicated "Parameter Definition" subsection in Methods section.

Revised parts in the manuscript)

- manuscript on page 19

"Parameter Definition

The sensitivity of the sensor reported in this work refers to the normalized sensitivity, defined as:

$$S = \frac{\Delta I/I_0}{\Delta P}$$

Where ΔI is the relative current variation, I_0 is the initial current without applied pressure, and ΔP is the applied pressure variation. the sensitivity is not constant over the entire pressure range due to the nonlinear contact evolution and conductive pathway reconstruction under compression. In this work, the reported sensitivity represents the average sensitivity within the 0-100 kPa pressure range, calculated from the slope of the best linear fitting of the corresponding pressure–response curve.

The linearity of the sensor was evaluated by fitting the measured pressure–response curve using the least-squares method. The coefficient of determination (R^2) of the linear fit was calculated to quantify the linearity."

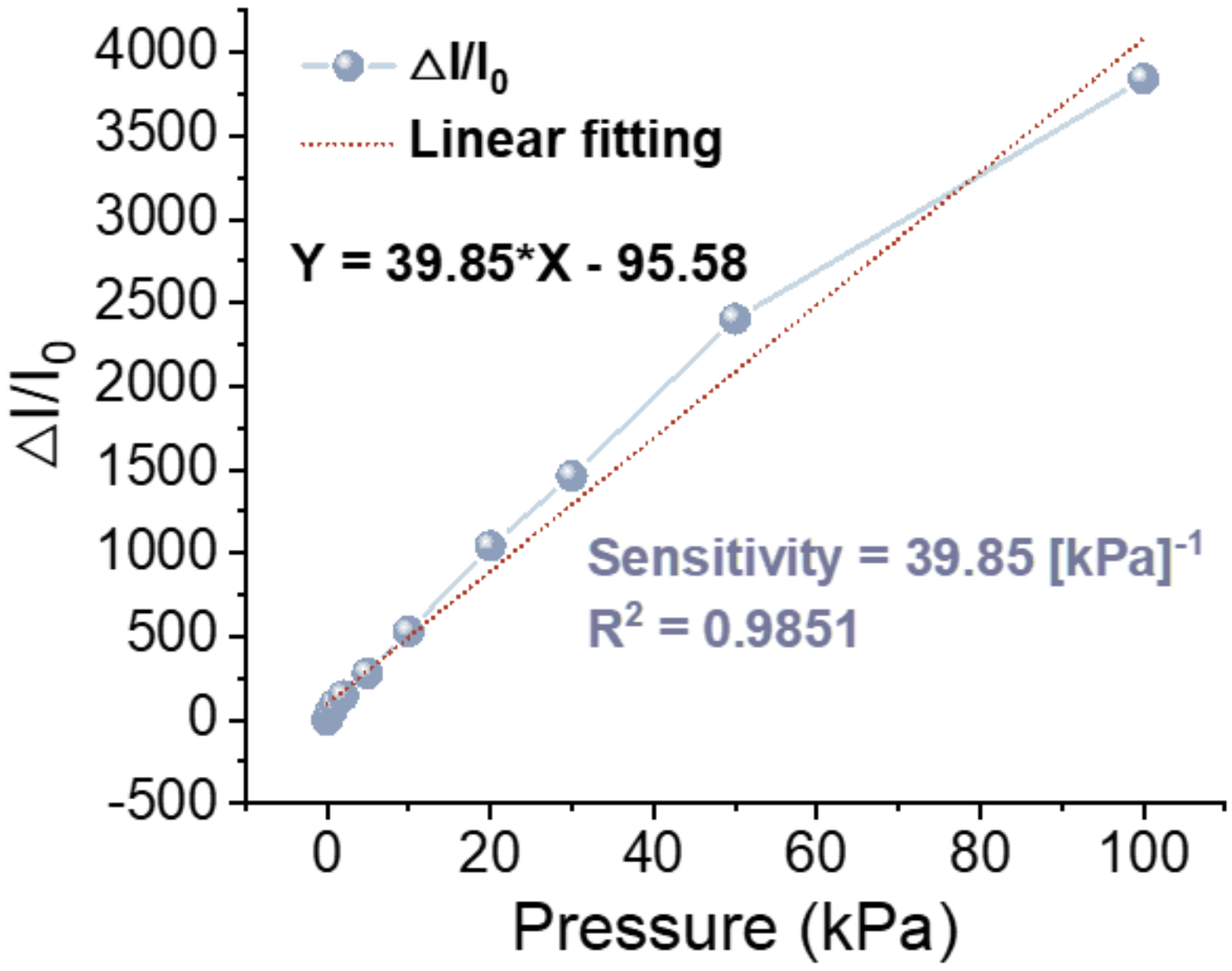

Figure 4. (b) Pressure response curve and linear fitting

Comment 3. *Line 182 (and in some other parts of the manuscript). Please highlight that 42 ms is the rise time (it can be confused with the time constant of the system).*

Response)

In this manuscript, the response time is defined as the time required for the output signal to increase from 0% to 90% of its steady-state value, also denoted as T_{90} (Figure 4d) [R9]. The rise time is similarly defined as the time for the output signal to increase from 10% to 90% of its steady-state value [R10]. Although the two terms have similar meanings, they are not entirely equivalent. In the sensor field, the use of response time is more common. For clarity and broader readability, we retain the term response time throughout the manuscript and have added a dedicated subsection in the Methods to explicitly define this parameter. The revised manuscript is as follows:

Revised parts in the manuscript)

- manuscript on page 19

"The response time was defined as the time required for the output signal to increase from 0% to 90% of its steady-state value (T_{90})."

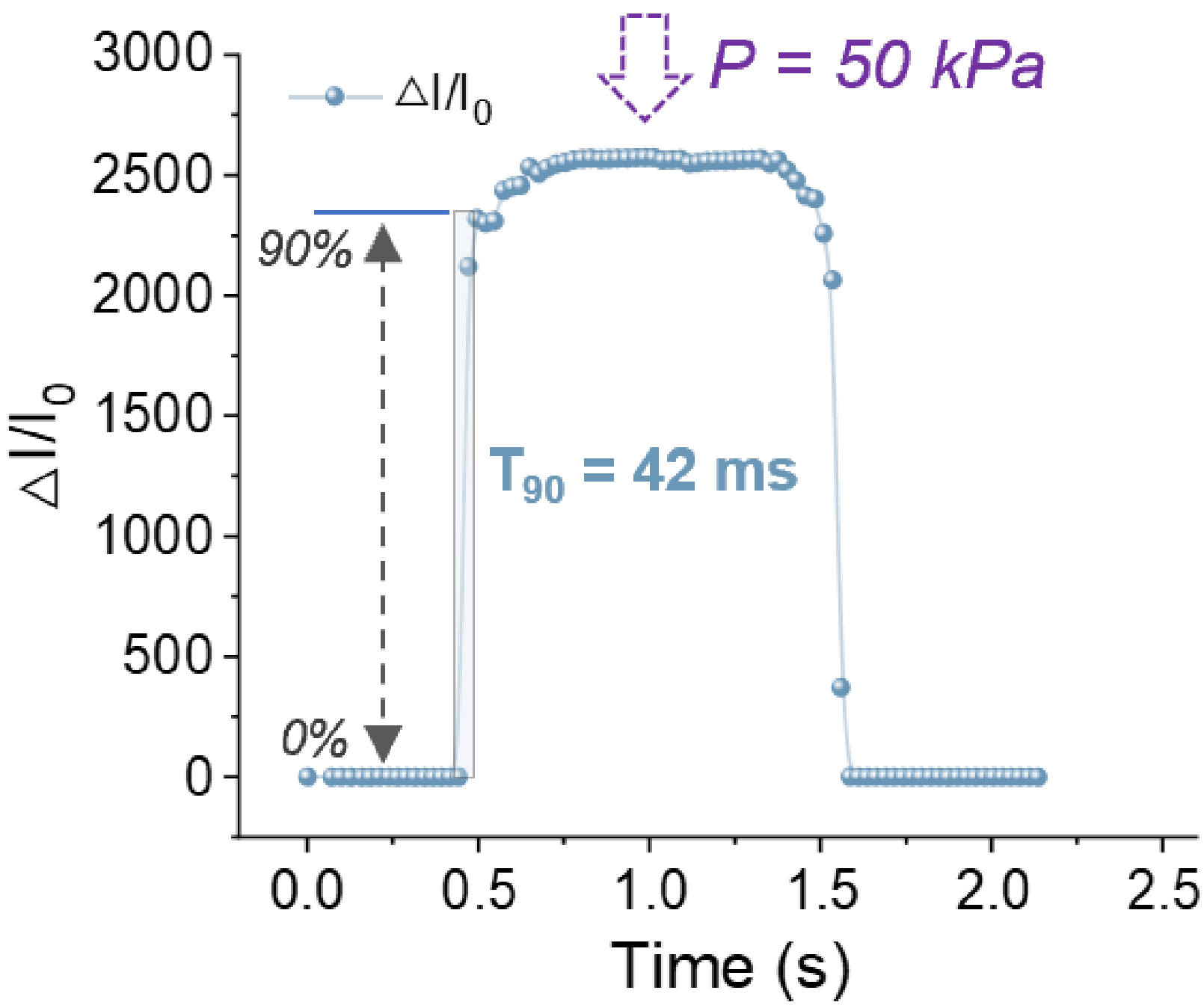

Figure 1. Comprehensive pressure-sensing performance of MASW. (d) Response time

Comment 4. *Line 205 (and in some other parts of the manuscript). Why did you consider a pressure range of 0-100 kPa? Is it motivated by the application of interest?*

Response)

We thank the reviewer for this insightful comment. The reviewer's assumption is correct. The selected pressure range of 0-100 kPa was determined based on practical application considerations. In wearable and physiological monitoring scenarios, typical pressures generated by human activities—such as pulse signals, finger pressing, gripping, joint bending, and plantar loading (see Table R1)—largely fall within this range. As summarized in the table, the 0-100 kPa range effectively covers nearly the full spectrum of physiological pressure signals encountered in the human body [R11].

Moreover, in view of the ultrahigh sensitivity of the MASW, we have specifically focused its application on the monitoring and evaluation of subtle yet physiologically significant signals, such as pulse waves and laryngeal movements, as well as on fine finger motion–controlled human–machine interaction systems. The 0-100 kPa detection range is more than sufficient to meet the requirements of these application scenarios.

Therefore, evaluating the device performance within 0-100 kPa ensures strong relevance to real-world applications.

Physiological activities	Pressure range
**Pulse (radial artery)**	0.5–5 kPa
**Carotid pulse / Swallowing**	1–10 kPa
**Light touch / Finger pressing**	5–50 kPa
**Object grasping**	10–100 kPa
**Joint bending**	1–50 kPa
**Plantar pressure (walking)**	50–300 kPa

Table R1. Typical Pressure Ranges Generated by Human Physiological Activities

Comment 5. *In the tables S2 and S3, you reported the response time that is different from the rise time. Please use the same parameter to compare the dynamic response of the proposed sensor with the sensors reported in the literature. This is crucial to compare your results with literature.*

Response)

We thank the reviewer for the careful reading. Since Table S2 primarily summarizes the linearity of pressure–response curves for MASWs fabricated under different process parameters, we believe the reviewer's concern regarding response time comparison likely refers to Tables S3 and S4.

As clarified in our response to **Comment 3**, the "response time" is defined as the time required for the output signal to increase from 0% to 90% of its steady-state value (T_{90}). This definition differs slightly from the reviewer's reference to "rise time", and an explicit explanation has been added to the Methods section. Notably, the literature used for performance comparison also reports dynamic behavior in terms of response time.

For consistency, we have adopted the term "response time" throughout the manuscript. We also noted that the unit was reported in seconds (s) in the tables but in milliseconds (ms) in the main text. To avoid confusion, we have unified both the terminology (response time) and the units (ms) throughout the revised manuscript and Supporting Information.

Revised parts in the manuscript)

- manuscript on page 19

"The response time was defined as the time required for the output signal to increase from 0% to 90% of its steady-state value (T_{90})."

Pressure-sensitive layer	Sensitivity [kPa]$^{-1}$	Response time (ms)	Linear operating range (kPa)	Pressure Cycles	Degradability	Reference
PSP	4.00	120	0-100	5000	×	Adv. Mater. 37, e2410312 (2025)
PVA/AU	0.03	220	0-100	1000	×	Science 370, 966-970 (2020)
PDMS:PVA	~10	660	~0-3	200	×	Nat. Commun. 16, 287 (2025)
EGaIn	0.11	640	0-12.5	-	×	Microsyst. Nanoeng. 11, 55 (2025)
P-TENG	9.21	70	0-8	2000	×	Nat. Commun. 16, 383 (2025)
prslPDMS	18.94	284	0-20	5000	×	Nat. Commun. 14, 1252 (2023)
PCM	0.57	192	1-13	600	×	Wearable Electron. 1, 180-188 (2024)
Paper/Ag	4.3	330	0-60	4000	√	npj Flex. Electron. 7, 13 (2023)
LS@CI-AgNPs	4.02	<00	0-50	1000	√	Small 21, e2407773 (2025)
PLA@CI-AgNWs	39.85	42	0-100	6000	√	This work

Table S3. Overall performance comparison of flexible pressure sensors, including sensitivity, response time, linear operating range, pressure cycles, and degradability.

Pressure-sensitive layer	Sensitivity $[kPa]^{-1}$	Response time (ms)	Degradability	Reference
PSP	4.00	120	×	Adv. Mater. 37, e2410312 (2025)
PVA/AU	0.03	220	×	Science 370, 966 -970 (2020)
PDMS:PVA	~10	660	×	Nat. Commun. 16, 287 (2025)
EGaIn	0.11	640	×	Microsyst. Nanoeng. 11, 55 (2025)
P-TENG	9.21	70	×	Nat. Commun. 16, 383 (2025)
prslPDMS	18.94	284	×	Nat. Commun. 14, 1252 (2023)
PCM	0.57	192	×	Wearable Electron. 1, 180 -188 (2024)
PDMS/Graphene	18.20	21	×	Adv. Funct. Mater., e10130 (2025).
PDMS/ AgNWs	0.018	468	×	Nat. Commun. 16, 2024 (2025).
Cellulose/MTS	11.95	93	√	Adv. Funct. Mater. 34, 2410762 (2024).
LS@CI-AgNPs	4.023	<300	√	Small 21, e2407773 (2025)
Paper/Ag	4.30	330	√	npj Flex. Electron. 7, 13 (2023)
POMaC	1.10	1500	√	Nat. Electron. 1, 314 -321 (2018)
CuNWs/chitosan	1.70	180	√	Org. Electron. 106, 106539 (2022)
AgNW	1.50	90	√	ACS Appl. Mater. Interfaces 11, 25034 - 25042 (2019)
Pectin xerogel	0.03	118	√	Adv. Funct.Mater. 34, 2403268 (2024)
PLA@CI-AgNWs	39.85	42	√	This work

Table S4. Comparison of sensitivity and response time of reported conventional and biodegradable flexible pressure sensors

Comment 6. *Line 282. The application of interest needs an accurate measure of pressure. Why did you use a voltage divider to convert R into V? When accurate measurements are required, it is much better to use a Wheatstone bridge.*

Response)

We thank the reviewer for this insightful comment. We agree that a Wheatstone bridge configuration generally provides higher accuracy for precise resistance measurements.

However, the primary application considered in this work is human–machine interaction (HMI), where real-time responsiveness, system simplicity, low power consumption, and ease of integration are prioritized over absolute metrological precision. The proposed sensor is intended for scalable and portable systems. Implementing a Wheatstone bridge would increase hardware complexity, calibration requirements, and power consumption, which are less favorable for compact wearable HMI platforms.

In addition, the developed HMI system is based on an Arduino Uno microcontroller, which directly acquires voltage rather than resistance signals. (Figure 6d) Therefore, a straightforward and efficient method is required to convert resistance variations of the sensor into measurable voltage changes.

In this context, a voltage divider configuration provides sufficient resolution to reliably distinguish different pressure levels while significantly reducing circuit complexity and power requirements.

The analog voltage was digitized using the built-in 10-bit ADC of the Arduino UNO microcontroller, which provided sufficient resolution for reliable discrimination of different pressure levels in HMI applications.

For applications requiring high-precision quantitative pressure measurement, a Wheatstone bridge configuration could be readily implemented; however, such precision is not required for the functional demonstration presented in this work.

A comprehensive comparison between the voltage divider and the Wheatstone bridge for HMI applications has been provided in Table R2. Considering the above factors, we adopted a voltage divider configuration rather than a Wheatstone bridge for pressure signal acquisition [R12].

Aspect	Voltage Divider	Wheatstone Bridge
**Measurement principle**	Converts resistance change directly into voltage variation	Measures differential voltage caused by resistance imbalance
**Measurement accuracy**	Moderate, sufficient for relative pressure sensing	High, suitable for precise resistance measurement
**Circuit complexity**	Very low (few passive components)	Higher (multiple resistors and balanced configuration)
**Calibration requirement**	Minimal	Requires careful balancing and calibration
**Power consumption**	Low	Relatively higher
**Signal conditioning**	Direct voltage output, easy ADC interfacing	Requires differential amplification
**Microcontroller compatibility**	Directly compatible with MCU ADC (e.g., Arduino)	Requires additional instrumentation amplifier
**System integration**	Highly suitable for compact and wearable systems	Less favorable for portable systems
**Hardware cost**	Low	Higher
**Application suitability**	Human–machine interaction, wearable sensing, real-time monitoring	High-precision metrology and instrumentation

Table R2. Comparison between Voltage Divider and Wheatstone Bridge for HMI Applications

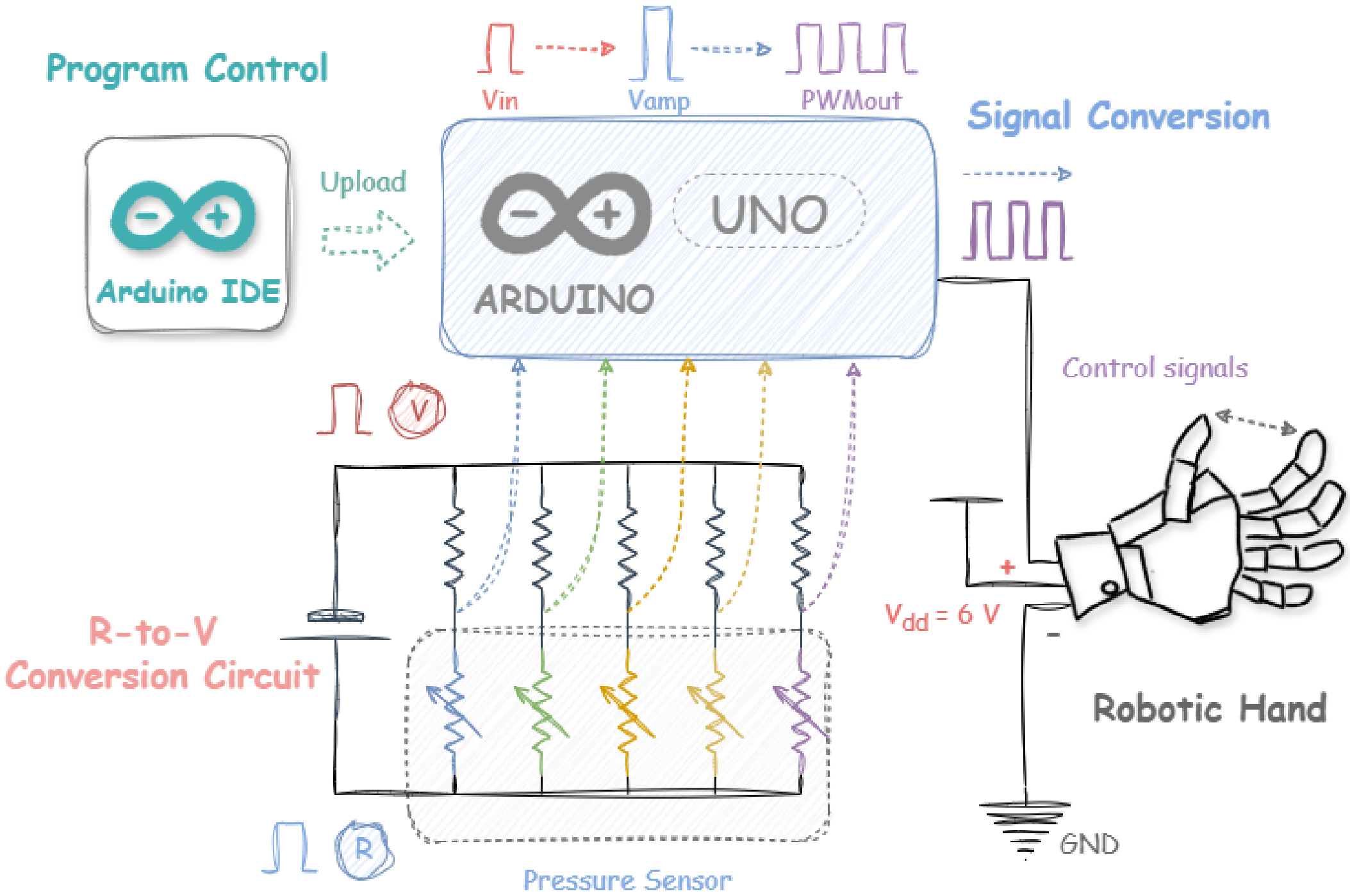

Figure 6. Human–machine interaction enabled by MASW. (d) Circuit diagram for MASW-controlled robotic hand

References

R1. Yoffe H. Modified Hertz theory for spherical indentation. *Philos. Mag. A* **50**, 813–828 (1984).

R2. Yuan H, Chen H, Li M, Liu L, Liu Z. Percolation threshold and electrical conductivity of conductive polymer composites filled with curved fibers. *Soft Matter* **19**, 7149–7160 (2023).

R3. Eugene M. The Early History of Quantum Tunneling. *Phys. Today* **55**, 44 (2002)

R4. Huang Q, et al. Electrochemical self-powered strain sensor for static and dynamic strain detections. *Nano Energy* **118**, 108997 (2023).

R5. Wei Q, et al. MXene-Sponge Based High-Performance Piezoresistive Sensor for Wearable Biomonitoring and Real-Time Tactile Sensing. *Small Methods* **6**, e2101051 (2022).

R6. Gao L, et al. All Paper-Based Flexible and Wearable Piezoresistive Pressure Sensor. *ACS Appl. Mater. Interfaces* **11**, 25034-25042 (2019).

R7. Li A, et al. All-Paper-Based, Flexible, and Bio-Degradable Pressure Sensor with High Moisture Tolerance and Breathability Through Conformally Surface Coating. *Adv. Funct. Mater.* **34**, 2410762 (2024).

R8. Wang Q, et al. Multimodal response characteristics of convective liquid metal sensitive layers in flexible pressure sensor. *Microsyst. Nanoeng.* **11**, 55 (2025).

R9. Dai J, Li H, Xie G, Que L, Yuan H, Su Y. Self-Supported Composite Textiles for Ultrasensitive Physiological Monitoring. *ACS Appl. Electron. Mater.* **7**, 7321-7329 (2025).

R10. Fraden, J. Handbook of Modern Sensors: Physics, Designs, and Applications. *Springer*, (2010).

R11. Lee Y, Park J, Cho S, et al. Flexible ferroelectric sensors with ultrahigh pressure sensitivity and linear response over exceptionally broad pressure range. *ACS Nano* **12**, 4045-4054 (2018).

R12. Horowitz P, Hill W. The Art of Electronics, *Cambridge University Press* (1989).
